# Convergent Evolution towards High Net Carbon Gain Efficiency Contributes to the Shade Tolerance of Palms (Arecaceae)

**DOI:** 10.1371/journal.pone.0140384

**Published:** 2015-10-13

**Authors:** Ren-Yi Ma, Jiao-Lin Zhang, Molly A. Cavaleri, Frank Sterck, Joeri S. Strijk, Kun-Fang Cao

**Affiliations:** 1 Key Laboratory of Tropical Forest Ecology, Xishuangbanna Tropical Botanical Garden, Chinese Academy of Sciences, Mengla, Yunnan, China; 2 University of Chinese Academy of Sciences, Beijing, China; 3 School of Forest Resources and Environmental Science, Michigan Technological University, Houghton, Michigan, United States of America; 4 Forest Ecology and Forest Management Group, Wageningen University, Wageningen, the Netherlands; 5 Plant Ecophysiology and Evolution Group, State Key Laboratory for Conservation and Utilization of Subtropical Agro-bioresources, and College of Forestry, Guangxi University, Nanning, Guangxi, China; INRA - University of Bordeaux, FRANCE

## Abstract

Most palm species occur in the shaded lower strata of tropical rain forests, but how their traits relate to shade adaptation is poorly understood. We hypothesized that palms are adapted to the shade of their native habitats by convergent evolution towards high net carbon gain efficiency (CGE_n_), which is given by the maximum photosynthetic rate to dark respiration rate ratio. Leaf mass per area, maximum photosynthetic rate, dark respiration and N and P concentrations were measured in 80 palm species grown in a common garden, and combined with data of 30 palm species growing in their native habitats. Compared to other species from the global leaf economics data, dicotyledonous broad-leaved trees in tropical rainforest or other monocots in the global leaf economics data, palms possessed consistently higher CGE_n_, achieved by lowered dark respiration and fairly high foliar P concentration. Combined phylogenetic analyses of evolutionary signal and trait evolution revealed convergent evolution towards high CGE_n_ in palms. We conclude that high CGE_n_ is an evolutionary strategy that enables palms to better adapt to shady environments than coexisting dicot tree species, and may convey advantages in competing with them in the tropical forest understory. These findings provide important insights for understanding the evolution and ecology of palms, and for understanding plant shade adaptations of lower rainforest strata. Moreover, given the dominant role of palms in tropical forests, these findings are important for modelling carbon and nutrient cycling in tropical forest ecosystems.

## Introduction

Palms are among the ecologically most important components of tropical rain forest ecosystems [1–4]. The palm family (Arecaceae) is the second most important family in terms of individual abundance in the Amazonian rainforest, especially palm species make up 6 of the 10 most abundant trees in the Amazon, and therefore they must account for a large proportion of ecosystem services, including carbon and nutrient cycling [5]. In a neotropical forest in Costa Rica palms even contribute 22% of total leaf area [6], making them an important functional group for estimating ecosystem respiration [7, 8]. Over 90% of palm species diversity is restricted to tropical rain forests [2] and the majority of them is primarily found in the light-limited forest understory and mid-storey [6, 9], suggesting that palms faced strong natural selection for shade tolerance. However, little is known about the mechanisms underlying such shade tolerance.

Two hypotheses have been put forward to explain the mechanisms underlying shade tolerance: the carbon gain hypothesis [10] defines shade tolerance as the maximization of net carbon gain in low light; and the stress tolerance hypothesis [11] defines shade tolerance as maximization of the resistance to biotic and abiotic stresses. However, there is a long-running debate on the first hypothesis, which centers on whether shade tolerance is mainly a function of traits minimizing carbon loss (e.g. low dark respiration, or slow leaf turnover) or of traits maximizing carbon gain in low light (reviewed by Valladares & Niinemets (2008) [12]). Furthermore, there is no mechanistic understanding linking these two hypotheses above, although Valladares and Niinemets (2008) [12] argued that these two mechanisms are not mutually exclusive.

It appears that palms have shade-tolerant traits supporting not only both alternative views of the carbon gain hypothesis but also the stress tolerance hypothesis. Cavaleri et al. (2008) [8] found that 12 palm species in an old-growth tropical rain forest possessed lower dark respiration than other functional groups such as trees and lianas. On the other hand, Chazdon (1986) [13] reported that understory palms often opportunistically took advantage of forest gaps and sunflecks to increase carbon gain potential, and consequently enhanced their growth and reproduction. Palms do not have a cambium for secondary growth, generally do not branch, and have limited numbers of large leaves. Therefore, palms must have leaf traits maximizing their resistance to biotic and abiotic agents. Their large and tough leaves contain high densities of fibers [14, 15] that reduce herbivory [16]. Large investments in support structures enable those leaves to resist mechanical damage [17]. As a result, palm leaves often have life spans of more than five years [18, 19], which is favorable for shade tolerance [12]. In addition, their primary growth organs, like root, stem and petiole, may store large amounts of nonstructural carbohydrates [18, 20, 21], which provide carbon and energy for growth and reproduction, and allow them to survive long periods of stress [22].

There may be a mechanism linking the two hypotheses explaining plant shade tolerance in palms. High carbon investment in the vast size of palm leaves, leaf toughness, herbivore defense and carbon storage for tolerating shade environments require a high carbon budget, which can be achieved by producing long-lived leaves [23–25]. However, carbon revenues may decrease in the long-term due to the time-discounting effect [23, 25], namely, due to ageing, overshading by surrounding vegetation and the plant itself, and injury by herbivores and falling canopy debris [23]. A high net carbon gain efficiency will be particularly favorable for such plants occurring in shaded habitats. Taking the ratio of the maximum leaf photosynthetic rate to dark respiration rate as a metric of net carbon gain efficiency (CGE_n_), natural selection should favor plants with high CGE_n_ either by minimizing respiration or by maximizing photosynthesis, especially in shady environments. High CGE_n_ would likely enable plants to allocate more to storage and thus facilitate the persistence in shady habitats [22]. Thus, the concept of CGE_n_ makes it possible to integrate alternative views of the carbon gain hypothesis and the stress tolerance hypothesis.

Natural selection combined with biophysical constraints would result in convergence in resource use strategies for different lineages [26]. The worldwide convergence in carbon and nutrient use strategies is described by the global leaf economics spectrum (LES), which reflects a pattern of correlated leaf traits across many lineages [24, 26, 27]. However, owing to their distinct characteristics, palms may differ in resource use strategy from other lineages. For example, their primary growth organs are capable of storing nutrients including nitrogen and phosphorus [20], which may be translocated to support photosynthesis when they are deficient in the soils. Palms are differ because they can keep a relatively constant number of open leaves in the crown through a highly constrained, regular process of leaf development, i.e. the new sword-leaf generally opens when the lowest (also oldest) leaf begins to die [28], during which the mobilizable nutrients (e.g. phosphorus) may be effectively resorbed and accumulated in new leaves [28]. How such distinctive traits relate to carbon and nutrient cycling is however poorly known. For example, only five palm species were included in the global LES dataset of Wright et al. (2004) [24], without data on photosynthesis or dark respiration.

Lineage-based studies of LES traits combined with the phylogenetic comparative method [29, 30] could yield valuable insights into the evolutionary adaptation and correlated evolutions between traits, and are vital to explain how ecological success has been achieved [31–34]. Using common garden plants, one is able to highlight genetically-based differences while minimizing environmental variation, thus trait values and relationships were anticipated to reflect adaptation to native habitats [32, 34]. The objective of this study is to characterize the carbon and nutrient economy in the context of phylogenetic relationships across the palm family. We hypothesized that palms are adapted to the shade of their native habitats by convergent evolution towards high net carbon gain efficiency. To test this hypothesis, we measured 80 palm species (S1 Table) from a palm live collection in a tropical botanical garden and compiled LES traits of 30 additional palm species from previous field studies (S2 Table). By comparing our palm datasets (common garden and field palms) with the global LES dataset containing a broad range of plant species from native habitats [24], and with datasets containing only dicotyledonous broad-leaved trees of tropical rain forests, or other monocotyledonous species, we could determine whether palms have their own phylogenetic (rather than common monocots) distinct leaf traits that would facilitate their adaptation to shade. Compared with other lineages, palms possessed lower dark respiration and higher CGE_n_, and had higher foliar P concentrations, supporting our stated hypothesis.

## Materials and Methods

### Study site and species

This study was carried out in the palm collection garden in Xishuangbanna Tropical Botanical Garden (21°41’N, 101°25’E, elevation 570 m) in southern Yunnan Province, China. Here, mean annual temperature is 21.7°C and mean annual precipitation is 1560 mm with 80% occurring in the rainy season (May-October). The soil of the palm garden was sandy alluvium, with pH of 6.0, containing 9.14 mg g^-1^ organic matter, 1.12 mg g^-1^ total N, 0.37 mg g^-1^ total P, 12.20 mg g^-1^ total K, and 88.40 mg kg^-1^ soil hydrolyzable N, 5.22 mg kg^-1^ available P, and 50.52 mg kg^-1^ available K at 0–20 cm depth, which is moderately fertile in N and very deficient in P [35]. The palms in this palm garden receive full sunlight, with photosynthetic photon flux density (PPFD) in the canopy reaching as high as 2000 μmol m^-2^ s^-1^. About 450 palm species have been collected since 1976 and all grow well in this palm garden.

Leaf traits were measured during the rainy seasons of 2011 and 2012. For the present study, 80 palm species from 78 genera in the common garden (dataset of common garden palms) were selected to maximize representation of major clades of the palm family, with six genera from subfamily Calamoideae, 30 genera from subfamily Coryphoideae, two genera from subfamily Ceroxyloideae, and 40 genera from subfamily Arecoideae, covering 17 of the 28 tribes of Arecaceae (S1 Table and S1 Fig). One subfamily (Nypoideae), which contains only one species occurring in mangrove forest, was not included in the palm garden collection. Although 24 palm species that occur naturally in high light conditions were included, most of our sample species (70%) occur naturally in low light conditions, which represent the shaded habitats that characterize the majority of species in the palm family (S1 Table). We also compiled LES traits of 30 palm species from previous field studies (dataset of field palms, [8, 9, 13, 16, 36–40]), covering 15 genera in 9 tribes (S2 Table). The same palm species in different studies (6 species) or study sites (1 species) were analyzed as independent data.

### Ethics Statement

The Horticulture Department of Xishuangbanna Tropical Botanical Garden gave permission to carry out this research and to collect leaf samples. We made our best effort to reduce injuries and other impacts to target species during the course of this research.

### Leaf gas exchange, leaf mass per area and nutrients

Six mature sunlit leaflets from three to six individuals per species were used for the measurements of leaf gas exchange, and a leaf sample was taken from the central part of a frond located second from the top. The sunlit leaves were chosen for the purpose of comparison with the global dataset. We are aware that sun leaves usually have higher photosynthesis and dark respiration on area basis than shade leaves [11, 41, 42]. The heights of sampled palms ranged from 0.5 to 13 m, and most palms (62 species) with height < 4 m (S1 Table) were measured using intact leaves, and 18 palm species were measured using harvested leaves because their leaves could not even be accessed with the help of a ladder. Area-based maximum photosynthetic rate (*A*_area_) was measured in the morning (08:30–11:30, solar time) during clear days and dark respiration was measured during the night (21:00–23:30), using a portable infrared gas analyzer (LI-6400, LI-COR, Nebraska, USA). For measurement of *A*_area_ from intact leaves, PPFD between 1000 and 1500 μmol m^-2^ s^-1^ was supplied to the leaf sample, which was photosynthetically saturated for 58 species. A lower PPFD (600–800 μmol m^-2^ s^-1^) was supplied during the measurement for the remaining four species, which were grown in partially shady conditions. For measurements from harvested leaves, leaflets attached to the rachis were cut down and recut under water, then illuminated with a PPFD of 1500 μmol m^-2^ s^-1^ to measure *A*_area_. All measurements were taken at a constant CO_2_ concentration of 390 μmol mol^-1^ in the reference chamber, at ambient temperature (26–35°C) and relative air humidity of 50–65%. The intact leaflets used for *A*_area_ measurement were tagged to measure dark respiration in the night, and the detached fronds used for measurement of *A*_area_ were covered with black plastic bags and transported to the laboratory for nighttime respiration measurements. Dark respiration was also measured at ambient temperature, and the leaf temperature was around 23±2°C, relative air humidity was 70–80%, and CO_2_ concentration of the reference chamber was maintained slightly higher than ambient air, keeping a small positive gradient in CO_2_ partial pressure for reliable estimation of respiration. We found that there were no differences in maximum photosynthetic rate and dark respiration between intact and detached leaflets (data not shown). Previous studies have also found no differences in respiration rates between intact and detached leaves in a range of species, including palms [8, 43]. We standardized the area-based dark respiration rate (*R*_area_) to a base temperature of 25°C using:

$$R_{area}=R_{Tleaf}\;\times \;Q_{10}^{\left[\left(25-Tleaf\right)÷10\right]}$$ (1)

where *R*_Tleaf_ is respiration rate at the measured leaf temperature, *T*_leaf_, and Q_10_ is the change in respiration rate with 10°C change in temperature. For palm species, the Q_10_ used in this analysis was 1.82, which was the average from four palm species measured in the field by Cavaleri et al. (2008) [8]. Net carbon gain efficiency (CGE_n_) was determined as *A*_area_ divided by *R*_area_.

The sections of the leaves used for gas exchange measurement or adjacent leaves were sampled during the next morning, and leaf area was determined with a portable area meter (LI-3000A, LI-COR, Nebraska, USA) with the thick central veins removed. The leaves were dried to constant weight at 70°C for 48 h. Leaf mass per area (LMA) was calculated as leaf dry mass divided by leaf area. Dry leaf samples were grounded and pulverized to pass a 60-mesh sieve. Total leaf N concentration (*N*_mass_) was determined using a carbon nitrogen analysis system (Vario MAX CN, Elementar Analysensysteme, Hanau, Germany). After the samples were digested with concentrated HNO_3_-HClO_4_, total leaf P concentration (*P*_mass_) was analyzed with an inductively coupled plasma atomic-emission spectrometer (iCAP6300, Thermo Fisher Scientific, MA, USA). Area- and mass-based traits were interconverted via LMA (for example, *A*_*mass*_ = *A*_*area*_ ÷ LMA). Mean values and ranges for these leaf traits of 80 common garden palms were shown in S3 Table.

### Data analysis

Statistical analyses were performed in R v 3.1.0 [44] and bivariate trait relationships were analyzed with Pearson’s (ahistorical) correlation. The differences in slope or intercept of bivariate relationships between common garden palms and plants from the global dataset [24], dicotyledonous trees in tropical rain forests (dicot TRF trees) or monocotyledonous species were examined with standardized major axis (SMA) tests using SMATR v2.0 [45]. The global dataset of leaf economics spectrum contained a broad range of plant species from native habitats (2548 species in total), but only five palm species without data of photosynthesis or dark respiration. The dataset of dicot TRF trees containing 231 species was compiled from the global dataset [24] and from later studies that provided leaf dark respiration rates ([40], 16 species; [41], 53 species; [46], 5 species; [47], 14 species) as also used in a recent global analysis of leaf dark respiration [48]. Leaf dark respiration rates from these references were normalized to 25°C according to Atkin et al. (2015) [48]. The dataset of monocots containing 130 species was compiled mainly from the global dataset [24] (127 species), and from [40] (1 species) and [46] (2 species). We also compared bivariate relationships of field palms with those of the global dataset and dicot TRF trees using SMATR v2.0 (S4 Table). To test whether the species that occur naturally in high light conditions biased the regression of the palm species that occur in low light conditions, we compared their bivariate trait relationships. Data were log_10_-transformed to satisfy the assumptions of normality and homoscedasticity.

To address the issue of statistical non-independence when analyzing closely related species, phylogenetic generalized least squares (PGLS) framework was used to test for bivariate traits relationships [30] in the R package APE [49] and nlme [50]. The branch lengths used in the PGLS analysis were obtained through phylogenetic reconstruction of common garden palm species. The phylogenetic tree (S1 Fig) was constructed using four chloroplast gene regions (rbcL, rps16, matK and trnL-trnF) and two nuclear gene regions (rpb2 and prk), and using Bayesian Inference analyses (S1 Method). DNA sequences were downloaded from published datasets of Baker et al. (2009, 2011) [51, 52] and GeneBank (http://www.ncbi.nlm.nih.gov/genbank), and were aligned according to Baker et al. (2009, 2011) [51, 52] into which new sequences were incorporated manually. With ambiguities (7 species) excluded, a phylogeny containing 73 palm species (each representing one genus) was highly bootstrap supported, and was in accordance with the well-resolved palm phylogeny at generic levels [51].

To statistically and visually assess the presence, strength and location of convergence in continuous trait data, we used several program packages and statistical indicators. To examine whether the shared evolutionary histories as specified by the phylogeny produce the patterns of similarity observed in the data, we tested the phylogenetic signal using the Pagel’s λ [53] implemented via PGLS framework [30]. We used the BEAST package (v1.8.2.-v2.0) [54] to simultaneously estimate phylogenetic relationships, reconstruct ancestral trait states and co-estimate continuous trait progression throughout our phylogeny (analyses details are placed in S2 Method). Tracer v1.6.0 [55] was used to analyze runs of BEAST and to check convergence. Finally, we used the R package SURFACE [56] to identify cases of convergent evolution. SURFACE uses the Hansen model of stabilizing selection around multiple adaptive peaks to infer a macro-evolutionary adaptive landscape using continuous trait data and a phylogenetic tree. The program uses stepwise AIC algorithms to fit a series of Hansen-models through a stepwise model-addition and -collapsing phase, leading to estimates of evolutionary convergence. We followed the manual provided by the authors, using default settings and compared our final constructed model against results obtained with simulated data sets under Brownian motion and Hansen null models, to evaluate whether convergence in this clade was greater than expected by chance alone.

## Results

### Correlated evolution of leaf traits across the palm family

**Fig 1:**
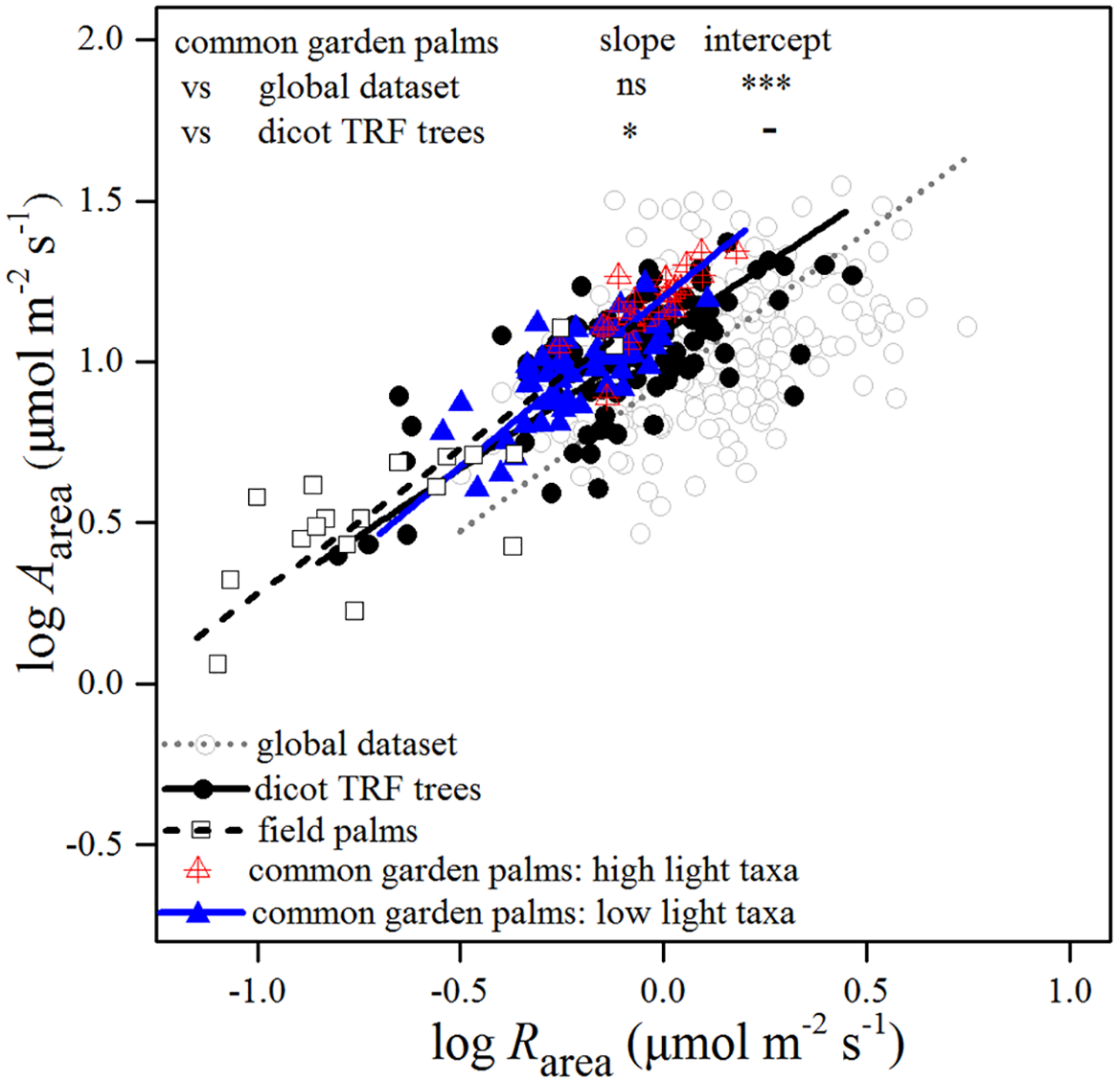
Relationships between area-based maximum photosynthetic rate (*A*_area_) and dark respiration rate (*R*_area_) across common garden palms, field palms, species from a global dataset and dicotyledonous broad-leaved trees in tropical rain forests (dicot TRF trees). Relationships in each dataset are significant and their standardized major axis (SMA) regression lines are shown. The differences in SMA regression slope and intercept between common garden palms and two other non-palm datasets are indicated (see S4 Table for the differences between field palms and two other non-palm datasets). ns, *P* > 0.05, *** *P* < 0.001.

All the 80 palm species grown in the common garden and all the field palm species we compiled exhibited consistently higher CGE_n_ than the other species from the global dataset and the dicot TRF trees (Fig 1). Palms had significantly higher *A*_area_ for any *R*_area_ as revealed by significantly higher regression intercept than for the global dataset and significantly higher regression slope than that for dicot TRF trees. Furthermore, the PGLS analysis confirmed the correlation between *A*_area_ and *R*_area_ in palms as this regression was still significant (P< 0.001, S5 Table). The other bivariate trait correlations fitted via PGLS framework were also significant (S5 Table) and consistent with their ahistorical correlations on both area and mass basis, suggesting correlated evolution between the examined leaf traits of palms.

**Fig 2:**
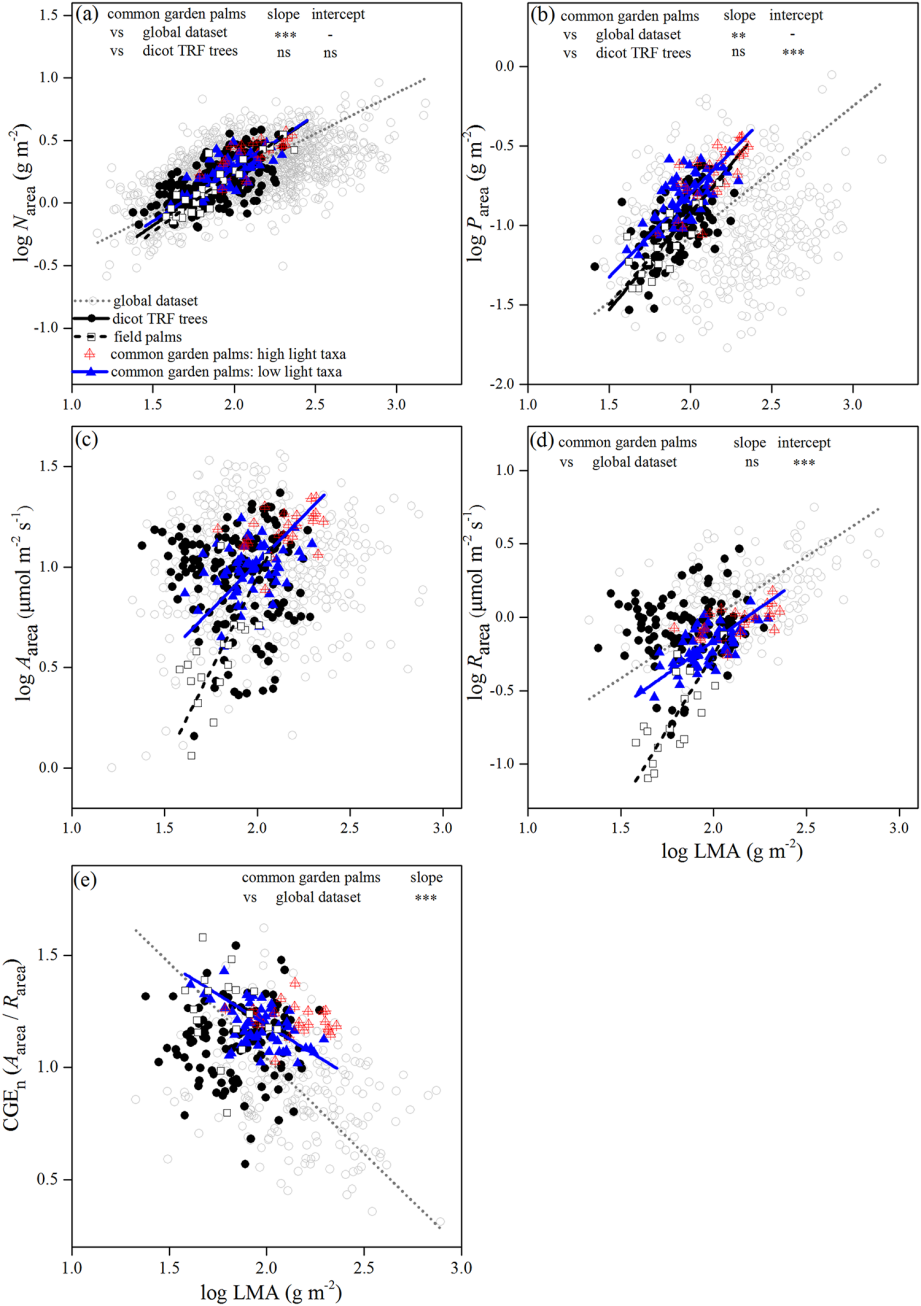
Trait relationships with leaf mass per area (LMA) as a variable across common garden palms, field palms, species from a global dataset and dicotyledonous broad-leaved trees in tropical rain forests (dicot TRF trees). (a) Area-based nitrogen concentration (*N*_area_), (b) area-based phosphorus concentration (*P*_area_), (c) area-based maximum photosynthetic rate (*A*_area_), (d) area-based dark respiration (*R*_area_), (e) net carbon gain efficiency (CGE_n_). Standardized major axis (SMA) regression lines are only shown for significant relationships. The differences in SMA regression slope and intercept between common garden palms and two other non-palm datasets are indicated (see S4 Table for the differences between field palms and two other non-palm datasets). ns, *P* > 0.05, ** *P* < 0.01, *** *P* < 0.001.

The bivariate correlations with LMA as a variable revealed that palms followed the same leaf functional design as the global leaf economics spectrum (Fig 2 and S2 Fig). Further, they revealed that the common garden palms had a *N*_area_ similar to dicot TRF trees (Fig 2a) but higher P than the global dataset and dicot TRF trees on both area and mass basis, and particularly so at high LMA (Fig 2b and S2b Fig). *A*_area_ in palms was positively correlated with LMA but not in the other species (Fig 2c). For a given LMA, palms exhibited significantly smaller *R*_area_ than species in the global dataset as revealed by smaller regression intercept and dicot TRF trees (Fig 2d). Consequently, for a given LMA, palms tended to have higher CGE_n_ than the global dataset and dicot TRF trees, and particularly so at high LMA (Fig 2e).

**Fig 3:**
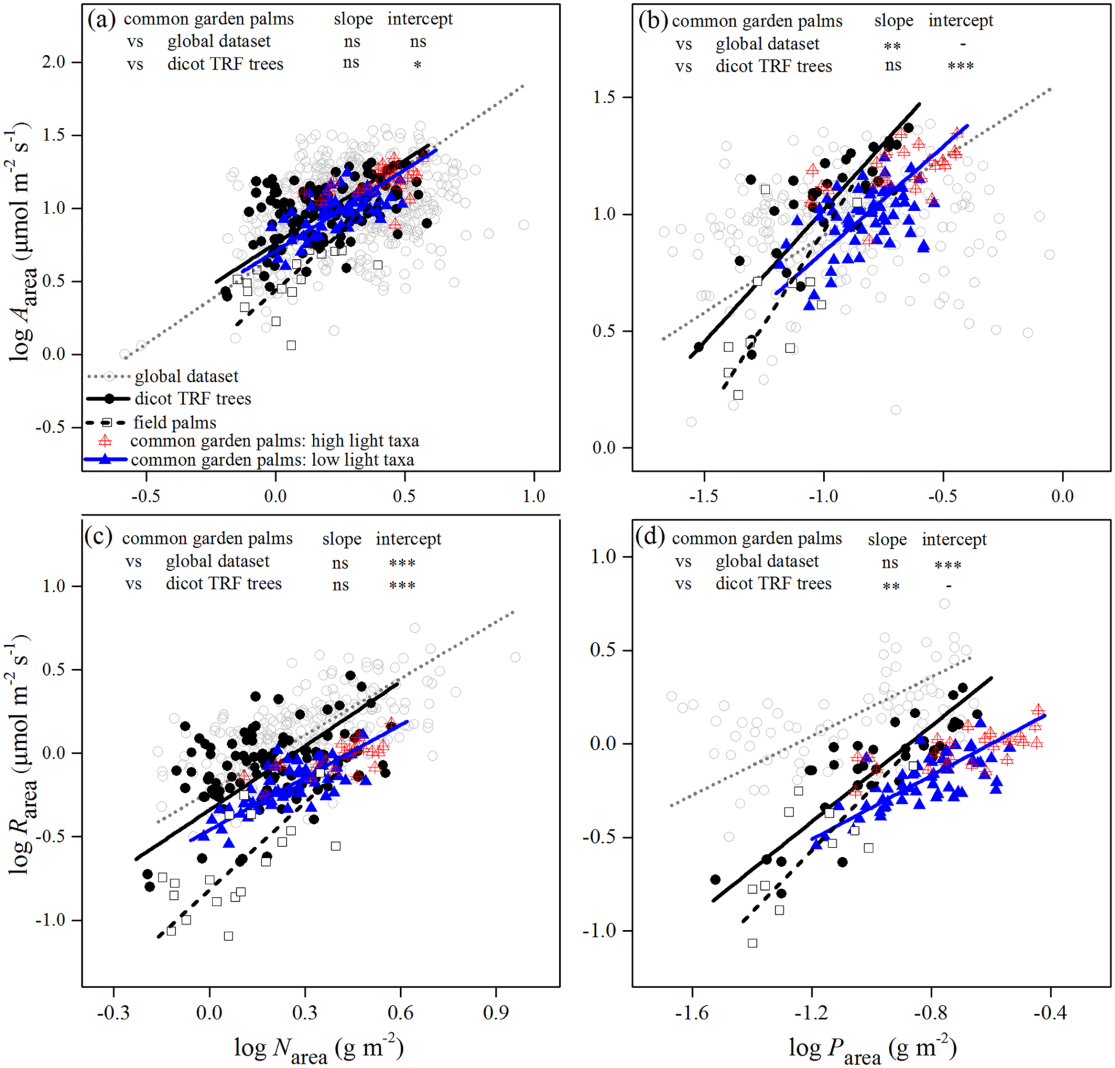
The dependency of photosynthesis and dark respiration on foliar area-based N (*N*_area_) and P (*P*_area_) across common garden palms, field palms, species from a global dataset and dicotyledonous broad-leaved trees in tropical rain forests (dicot TRF trees). (a, b) Area-based maximum photosynthetic rate (*A*_area_), (c,d) area-based dark respiration rate (*R*_area_). Relationships in each dataset are significant and their standardized major axis (SMA) regression lines are shown. The differences in SMA regression slope and intercept between common garden palms and two other non-palm datasets are indicated (see S4 Table for the differences between field palms and two other non-palm datasets). ns, *P* > 0.05, * *P* < 0.05, ** *P* < 0.01, *** *P* < 0.001.

The dependence of photosynthesis and dark respiration on foliar N and P nutrients in palms followed the general global trend (Fig 3 and S3 Fig). Common garden palms possessed area-and mass based maximum photosynthetic rates that were similar to those rates for the species from the global dataset (Fig 3a and S3a Fig). However, palm species of the common garden and native habitats showed a significantly lower area-and mass-based dark respiration compared to the other species, particularly so in the field palms. The intercept of the regression between *R*_area_ and *N*_area_ for palms was significantly lower than for the global dataset and dicot TRF trees (Fig 3c), and the slope of the regression between *R*_mass_ and *N*_mass_ was significantly lower for palms than for other two datasets (S3c Fig). Moreover, comparing the regression between dark respiration and foliar P both on area and mass basis, the common garden palms also had a much lower regression intercept than the global dataset and lower regression slope than dicot TRF trees (Fig 3d and S3d Fig).

Most of the LES traits of field palms varied within the ranges of the common garden palms (Figs 1–3; S2 and S3 Figs). Although some of them extended to the lower value ranges of trait combinations, the palms in native habitats still showed a significantly higher *A*_area_ at a given *R*_area_ and higher *P*_area_ at a given LMA than the global dataset, and this difference became stronger at high LMA (Figs 1 and 2b; S4 Table). They also possessed lower *R*_area_ at a given LMA, *N*_area_ and *P*_area_ than the global dataset and dicot TRF trees (Figs 2d, 3c and 3d; S4 Table).

**Fig 4:**
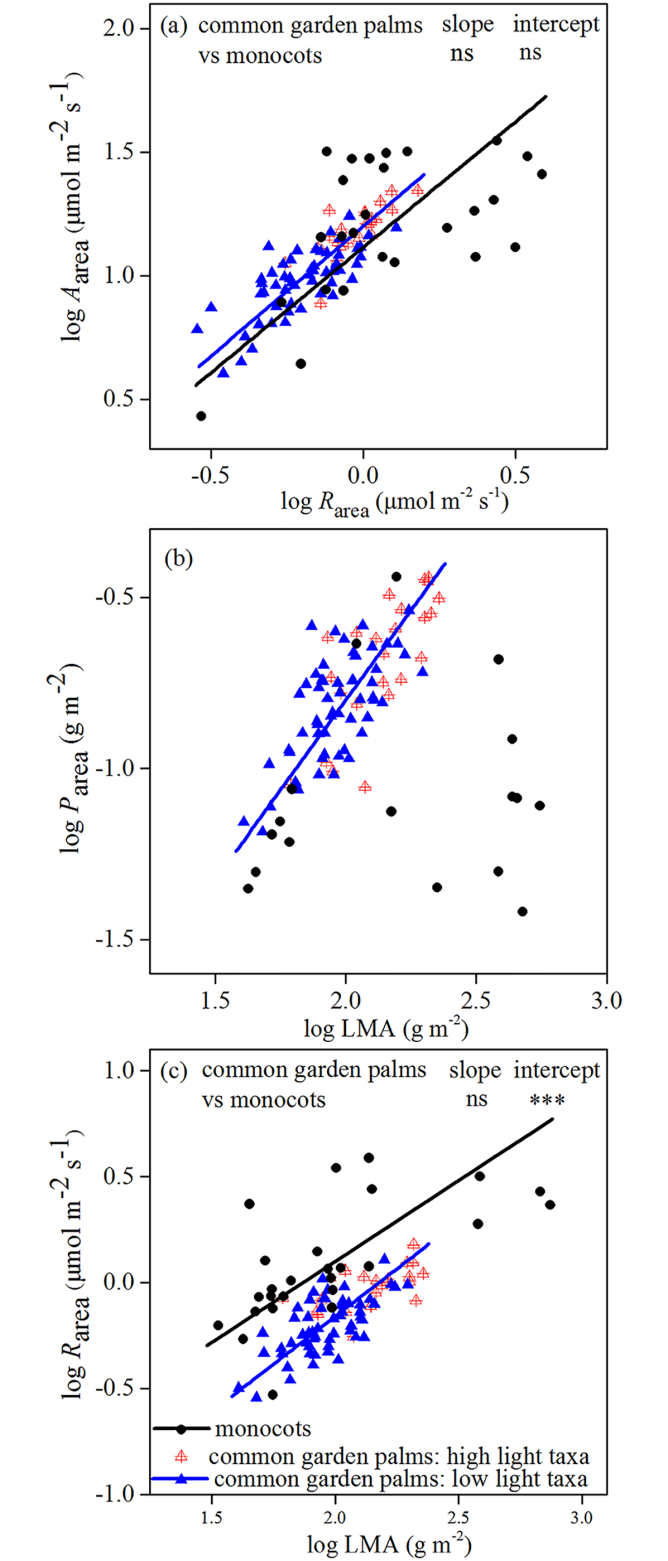
Trait relationships across common garden palms and other monocots. (a) Area-based maximum photosynthetic rate (*A*_area_) versus dark respiration rate (*R*_area_), (b) area-based phosphorus concentration (*P*_area_) versus leaf mass per area (LMA), (c) and *R*_area_ versus LMA. Standardized major axis (SMA) regression lines are only shown for significant trends. The differences in SMA regression slope and intercept between common garden palms and other monocots are indicated. ns, *P* > 0.05, *** *P* < 0.001.

The bivariate trait relationships of the palm species that occurred naturally in high-light and low-light conditions followed the same regression lines (Figs 1–4). However, the high-light taxa were in the high ranges of trait combinations. Compared with other monocots, common garden palms tended to have a higher *A*_area_ at a given *R*_area_, although regression coefficients were not significant (Fig 4a). Palms also possessed higher *P*_area_ and lower *R*_area_ for a given LMA than other monocots (Fig 4b and 4c).

### Phylogenetic analyses

The phylogenetic signals in all traits were weak, with Pagel’s λ < 1 (S6 Table), indicating that the traits of the closely related palm species were less similar to each other than expected from their phylogenetic relationships and were evolutionarily malleable. Pagel’s λ were higher for the five area-based traits than the mass-based traits, revealing a high degree of evolutionary malleability in area-based LES traits in this lineage.

**Fig 5:**
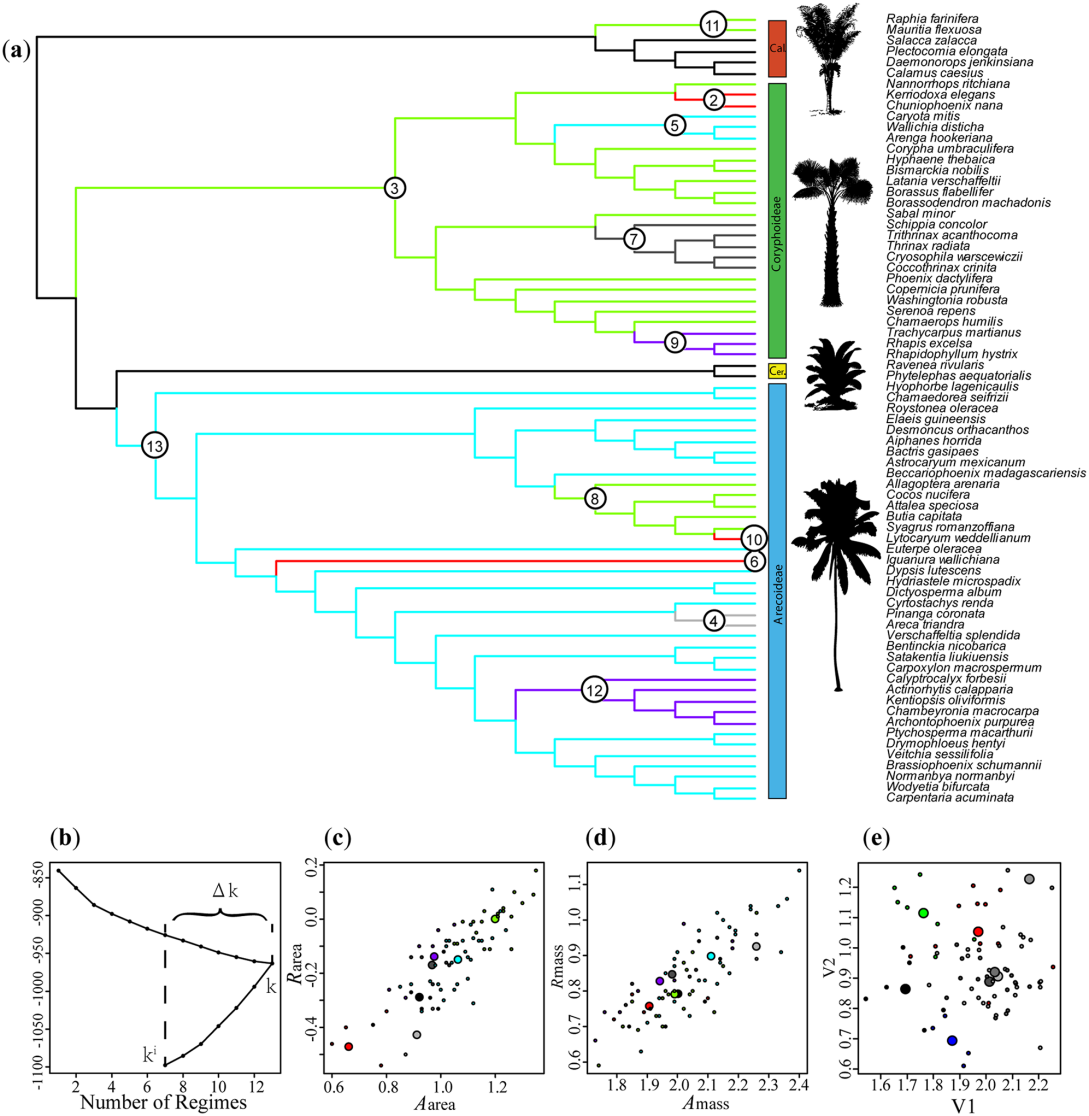
Results of a SURFACE analysis of the palm family. (a) Phylogenetic tree (MCC obtained in BEAST), with surfaceTreePlot painting to highlight convergent (colored) and non-convergent (black/grey) regimes onto branches. Numbers on branches indicate the order in which regime shifts were added during the forward phase. (b) AIC_c_ plot showing Δk and AIC-changes during forward and backward phases of the SURFACE analysis. (c) and (d) Comparison of convergence between area-based and mass based convergence (*A*_area_ vs. *R*_area_, and *A*_mass_ vs. *R*_mass_ respectively). (e) Results obtained using the null expectation under a non-convergent Hansen model. Note for (c-e) Plots showing trait values for species (small circles) and estimated trait optima (large circles). Regime colors match those used in (a). Palm illustrations from Phylopic.org.

Combined analyses of continuous trait data and DNA sequence data did not modify the previously obtained phylogeny of DNA sequence analyses with MrBayes (trees shown in Fig 5 and S1 Fig). Convergence was checked using Tracer and we found effective sample sizes of every parameter exceeding 100. To visually assess the difference in progression and quantitative change of ancestral states of traits throughout the palm family, each trait was highlighted individually onto the obtained maximum clade credibility tree (MCC-tree, S4 Fig). Obtained co-reconstructions for LMA, *A*_area_, *R*_area_ and *N*_area_ gave highly similar results, with most major increases in recently diverged Coryphoideae. Arecoideae showed simultaneous decreases in several lineages. CGE_n_ is fairly stable throughout the phylogeny, except for a few more recently diverged clades in Coryphoideae and Arecoideae. Mass-based traits showed less widespread increases when compared to area-based trait data (S4 Fig), confirming the results of our phylogenetic signal test (S6 Table).

The Hansen model obtained in our SURFACE analysis included 13 regime shifts, with seven distinct regimes (Δk = 6) and c = 10 convergent shifts (Fig 4). AIC_c_ improved from -840.2 to -962.9 (ΔAIC_c_ = 122.7) during the forward phase, with a final AIC_c_ of -1097.9 during the backward phase (ΔAIC_c_ = 135.0). Four convergent regimes were included in the final model, with all except one of them, reached by a single regime shift in the four subfamily clades (except for Ceroxyloideae, which did not receive a regime assignment). A major phylogenetic division in regimes is visible between the two main subfamilies of palms in our phylogeny (i.e. Arecoideae vs. Coryphoideae). However, in several minor clades, shifts to dominant or minor regimes of the other major subfamily occurred (e.g. Corphoideae: 5; Arecoideae: 8; both subfamilies: 12 and 9; 6, 10 and 2). Comparison of our results against those obtained under a non-convergent Hansen model shows stronger convergence than expected by chance. Convergence appeared less strong in mass-based than in area-based trait data sets.

## Discussion

This study presents the first systematic survey of leaf functional traits and resource use strategies within the palm family. Combining the palm data from 80 species grown in a common garden with 30 species in native habitats, strong evidence was found that palms have high net carbon gain efficiency, a convergent evolutionary strategy that allow palms to adapt to shade habitats. Although there were 24 palm species sampled that occurred naturally in high light conditions, they didn’t bias the bivariate trait relationships of the low light palm species. By comparing our common garden palms with other monocotyledonous species, we found that high net carbon gain efficiency, high foliar P concentration and low dark respiration were characteristic for palms only, and not a generic monocot phenomenon.

Our results reveal that high net carbon gain efficiency in palms resulted from convergent evolution across the palm family (Figs 1 and 5), with distinctly higher CGE_n_ than other plant species including broad-leaved trees of tropical rain forests (Figs 1 and 2e). High net carbon gain in understory plants would allow a fairly large allocation of carbohydrates to storage, which should be favorable for the palm species to grow in their native habitats of the tropical forest understory [22]. In addition, plants with higher CGE_n_ could obtain more carbon at a given time, especially when light condition in the forest understory is improved. In line with this idea, understory palms were found to be more efficient in using forest gaps and sunflecks to increase carbon gain potential [13]. This provides additional advantage to palms in competition with trees in the understory of tropical rain forests. Furthermore, high CGE_n_ could compensate for the reduced carbon revenue caused by the time-discounting effect [23], especially for the long-lived palm leaves of which there are generally fewer and thus more valuable than those of dicotyledonous trees. High net carbon gain could enable palms to invest a sizable carbon fraction to construct their tough and large leaves to reduce losses to herbivores [16] and mechanical damage [13]. Palms have notably high densities of fibres in the lamina [14, 15] and their leaf losses due to herbivores are much smaller than for dicots in tropical rain forests [16]. As a consequence, high CGE_n_ may enable palms to better adapt to shady environments than coexisting dicot tree species.

High CGE_n_ across palms is mainly attributed to commonly low dark respiration, as found in the palm species in the common garden and in the field (Fig 2d and S2d Fig). Low dark respiration is beneficial for a plant in a shady environments, because it allows a plant to maintain a positive carbon balance under conditions where carbon assimilation is limited by light availability. In such case, low dark respiration would be a target of natural selection [43]. Consistent with our findings in palms, other studies have shown that shade-tolerant species and plants grown at lower-irradiance sites usually had lower dark respiration than less-tolerant species and plants at higher-irradiance sites [11, 40, 41, 57]. Cavaleri et al. (2008) [8] also found that the twelve palm species measured in an old-growth tropical rain forest had lower dark respiration than trees and lianas. In addition, commonly low dark respiration across palms may also be due to their restricted distribution to the tropics and subtropics [1, 3], where plants exhibited lower dark respiration than the species in cold habitats [48]. Remarkably, however, dark respiration is generally even lower in palms than in the dicot trees (Fig 2d) with whom they compete in tropical rain forest understories.

Our results also revealed high leaf P concentration in palms (Fig 2b and S2b Fig), which may potentially contribute to low respiration cost (Fig 3d and S3d Fig) and high CGE_n_ through enhancing exportation of photosynthate to non-photosynthetic tissues. Phosphorus regulates photosynthetic rate by facilitating newly assimilated carbohydrate to be exported from chloroplasts to the cytosol as triose-P [58], and provides a driving force for ATP consumption for the exportation of newly assimilated carbon to other parts of plants (e.g. carbon reserve organs) through the apoplastic pathway [59]. Hence, high leaf P facilitates the production and exportation of photosynthate, while at the same time, decreasing the substrate for respiration and facilitating storage of carbohydrate. So what enables palms to accumulate high leaf P in the P deficient soils of both the common garden and native habitats? First, arbuscular mycorrhizal fungi have been found in all the palm species examined, which could enhance the uptake of P especially when the soil is P deficient [60]. Secondly, the stored P in the stem [20] may provide a constant supply of P to palm leaves. Finally, the highly constrained, regular process of leaf development could facilitate P to be effectively resorbed and accumulated in new leaves [28]. We found that the resorption efficiency in nine palm species ranged from 65% to 92% with an average of 75% (RY Ma, unpublished data). This is much higher than the eight *Banksia* species that occur on the most P-impoverished soils in the world and have very high P resorption efficiency (53.5%; [58]). Edwards et al. (2010) [28] also found that the P resorption efficiency in *Lodoicea maldivica* palm was more than 90%. In addition, they also described a mechanism for palms to improve nutrient supply: palm leaves could form a huge funnel to channel nutrient-rich particulate material to the base of the plant.

Palms have long-lived, tough and large leaves, strongly preventing herbivory [1, 14, 16]. Therefore, palm species have shade-tolerant traits that support both the carbon gain hypothesis and stress tolerance hypothesis. The mechanism linking those hypothesis is that palms have a high CGE_n_ by minimizing dark respiration and that high CGE_n_ enables palms to store and allocate more carbon to leaf structural materials so as to resist biotic and abiotic stresses. The understory palm species *Geonoma cuneata*, which can exploit the shadiest microsites within a rain forest [13, 61], had the highest CGE_n_ (S2 Table), thus underlining that a high CGE_n_ contributes to shade tolerance.

Although palm species differ in net carbon gain efficiency, they still follow the general functional design between leaf traits as described by the global leaf economics spectrum. The tight correlations between leaf carbon and nutrient economic traits in palms were also supported by PGLS analyses (S5 Table), implying that these leaf traits are linked to the evolutionary development of the family. All these traits showed weak phylogenetic signals with Pagel’s λ < 1 (S6 Table), indicated that they were highly adaptive [53] in the environment where palms persisted. The result that area-based traits are more evolutionarily malleable than mass-based traits is also an advantage for palms to adapt to shady environment, where natural selection should favor plants that supply a maximal photosynthetic surface area to intercept light and capture CO_2_ with a minimal mass cost [13]. Under natural selection, the correlated evolution of these palm traits results in a convergent higher CGE_n_ than other species, enabling them to better adapt and persist in shady environments. In addition, results from our SURFACE analyses seem to indicate that different subfamilies of palms find overall different optimal adaptive regimes (with the exception of some minor clades). This suggests the existence of different LES-trait-optima which are closely linked to leaf-design, leaf-morphology and palm-habit, as there are broad differences in these properties between the different subfamilies used in this study (Calamoideae: climbing or non-climbing palms with mostly pinnate leaves; Coryphoideae: small to medium sized palms with palmately divided leaves; Ceroxyloideae: small to giant palms with pinnate, entire or bifid leaves; Arecoideae: minute to large palms with pinnate of bipinnate leaves).

In conclusion, our data provide strong support for the hypothesis that high net carbon gain efficiency results from convergent evolution for shade adaptation of palms. Low dark respiration and high leaf P concentration of long-lived tough leaves are distinct leaf traits for palms, and may convey advantages in competing with coexisting dicot tree species in the shaded tropical forest understory. These findings provide important insights for understanding the evolution and ecology of palms, and for understanding plant shade adaptations of lower rainforest strata. They also add to our knowledge on the leaf economics spectrum of the plant family Arecaceae, a key family in tropical forests, which is important information for modelling carbon and nutrient cycling in tropical forest ecosystems.

## Supporting Information

S1 Fig Evolutionary relationships among the 73 palm species included in phylogenetic independent contrasts analysis. (DOCX)

S2 Fig Mass-based trait relationships with leaf mass per area (LMA) as a variable across common garden palms, field palms, global dataset and dicotyledonous broad-leaved trees in tropical rain forests. (DOCX)

S3 Fig Trait relationships among mass-based photosynthesis, dark respiration and nutrients across common garden palms, field palms, global dataset and dicotyledonous broad-leaved trees in tropical rain forests. (DOCX)

S4 Fig Maximum clade credibility (MCC) tree obtained with Beast analysis of DNA and multivariate trait partitions. (DOCX)

S1 Method DNA sequence alignment and phylogenetic reconstruction of palm species grown in the common garden. (DOCX)

S2 Method Coevolutionary patterns of morphological traits and ancestral state reconstruction with simultaneous phylogenetic reconstruction. (DOCX)

S1 Table Description of the study palm species grown in common garden with their GenBank accession numbers for DNA sequences. (DOCX)

S2 Table Compiled data on leaf traits of field palm species. (DOCX)

S3 Table Mean values and ranges for 10 leaf traits of 80 common garden palm species. (DOCX)

S4 Table Differences in slope and intercept of bivariate relationships of field palms with those of the global dataset and dicotyledonous broad-leaved trees in tropical rain forests (dicot TRF trees). (DOCX)

S5 Table Correlation coefficients *r* and *P* values for each bivariate trait relationship fitted via phylogenetic generalized least squares (PGLS) framework. (DOCX)

S6 Table Results of the phylogenetic signal tests for area-based and mass-based LES traits among palm species (DOCX)
