# Supplementary material for: Convergent Evolution towards High Net Carbon Gain Efficiency Contributes to the Shade Tolerance of Palms (Arecaceae)
Source: PLoS One. 2015 Oct 13;10(10):e0140384. doi: 10.1371/journal.pone.0140384 (PMC4604201; doi:10.1371/journal.pone.0140384)
Supplement: S1 Fig — (DOCX) [file pone.0140384.s001.docx]

**
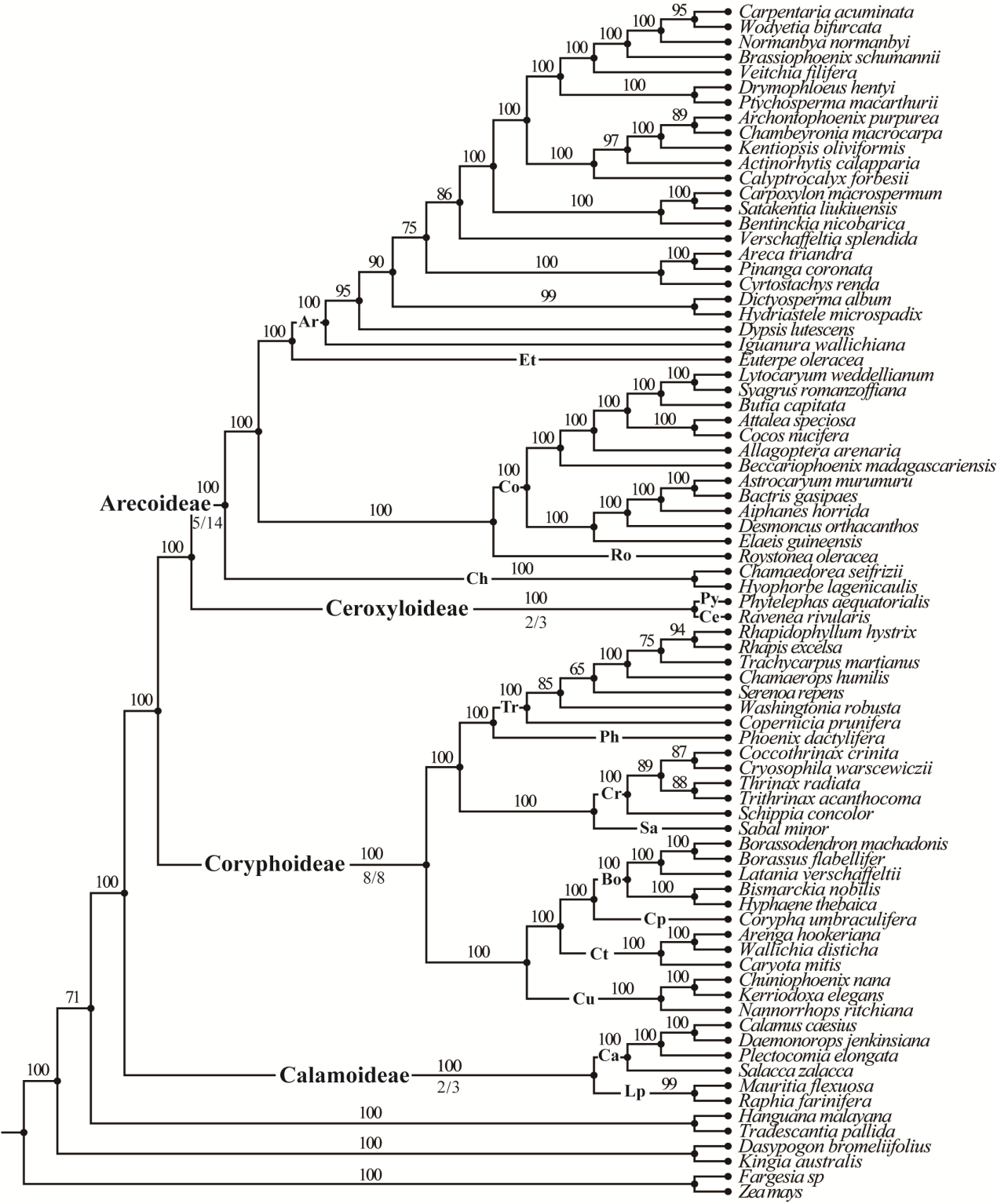
**

**S1 Fig. Evolutionary relationships among the 73 palm species included in phylogenetic independent contrasts analysis.** Phylogenetic analyses were conducted using four chloroplast gene regions (rbcL, rps16, matK and trnL-trnF) and two nuclear gene regions (rpb2 and prk), using Bayesian Inference analyses. Values above the branches are bootstrap percentages (>50%). Values below branches indicate the number of tribes included in this study/total number of tribes in that subfamily. Palm subfamilies and tribes are indicated. Key to abbreviations, Ar, Areceae; Bo, Borasseae; Ca, Calameae; Ce, Ceroxyleae; Ch, Chamaedoreeae; Co, Cocoseae; Cp: Corypheae; Cr, Cryosophileae; Ct, Caryoteae; Cu, Chuniophoeniceae; Et, Euterpeae; Lp, Lepidocaryeae; Ph, Phoeniceae; Py, Phytelepheae; Ro, Roystoneeae; Sa, Sabaleae; Tr, Trachycarpeae.
