# Supplementary material for: Convergent Evolution towards High Net Carbon Gain Efficiency Contributes to the Shade Tolerance of Palms (Arecaceae)
Source: PLoS One. 2015 Oct 13;10(10):e0140384. doi: 10.1371/journal.pone.0140384 (PMC4604201; doi:10.1371/journal.pone.0140384)
Supplement: S2 Fig — (DOCX) [file pone.0140384.s002.docx]

**S2 Fig. Mass-based trait relationships with leaf mass per area (LMA) as a variable across common garden palms, field palms, global dataset and dicotyledonous broad-leaved trees in tropical rain forests (dicot TRF trees)**. (a-d) mass-based nitrogen concentration (*N*_mass_), phosphorus concentration (*P*_mass_), maximum photosynthetic rate (*A*_mass_), dark respiration (*R*_mass_) plotted against LMA. Data were fitted by standardized major axis (SMA) regression and the differences in SMA regression slope between common garden palms and two other non-palm datasets are indicated. There were no significant correlations in all relationships in field palms. * *P* < 0.05, ** *P* < 0.01, *** *P* < 0.001.
