# Supplementary material for: Convergent Evolution towards High Net Carbon Gain Efficiency Contributes to the Shade Tolerance of Palms (Arecaceae)
Source: PLoS One. 2015 Oct 13;10(10):e0140384. doi: 10.1371/journal.pone.0140384 (PMC4604201; doi:10.1371/journal.pone.0140384)
Supplement: S3 Fig — (DOCX) [file pone.0140384.s003.docx]

**S3 Fig. Trait relationships among mass-based photosynthesis, dark respiration and nutrients across common garden palms, field palms, global dataset and dicotyledonous broad-leaved trees in tropical rain forests (dicot TRF trees)**. (a) between mass-based maximum photosynthetic rate (*A*_mass_) and nitrogen concentration (*N*_mass_); (b) between *A*_mass_ and phosphorus concentration (*P*_mass_); (c) between mass-based dark respiration (*R*_mass_) and *N*_mass_; (d) between *R*_mass_ and *P*_mass_. Data were fitted by standardized major axis (SMA) regression and the differences in SMA regression slope and intercept between common garden palms and two other non-palm datasets are indicated. There were no significant correlations in all relationships in field palms. ns, *P* > 0.05, ** *P* < 0.01, *** *P* < 0.001.
