# Supplementary material for: Convergent Evolution towards High Net Carbon Gain Efficiency Contributes to the Shade Tolerance of Palms (Arecaceae)
Source: PLoS One. 2015 Oct 13;10(10):e0140384. doi: 10.1371/journal.pone.0140384 (PMC4604201; doi:10.1371/journal.pone.0140384)
Supplement: S4 Fig — (DOCX) [file pone.0140384.s004.docx]

**
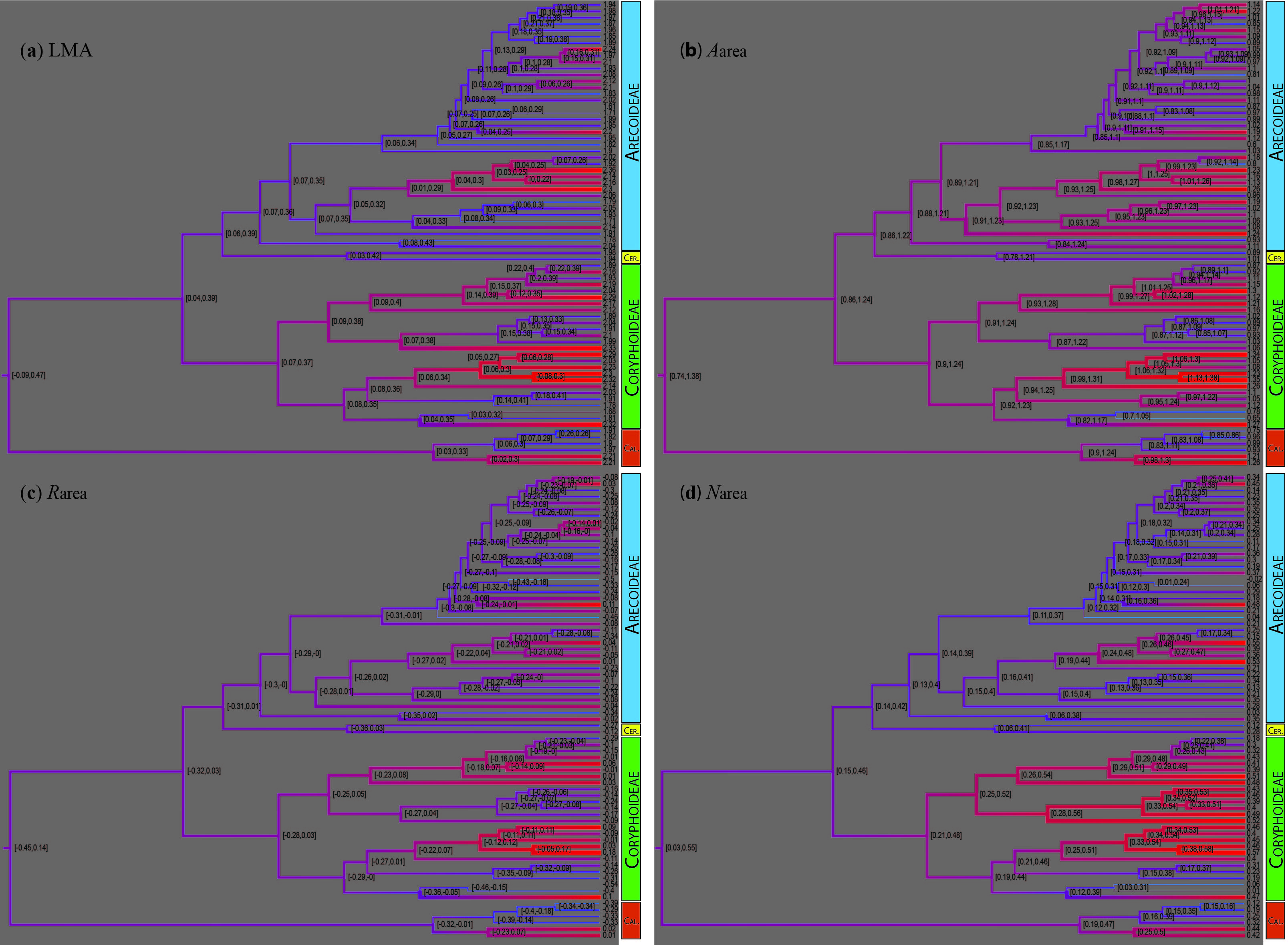
**

**
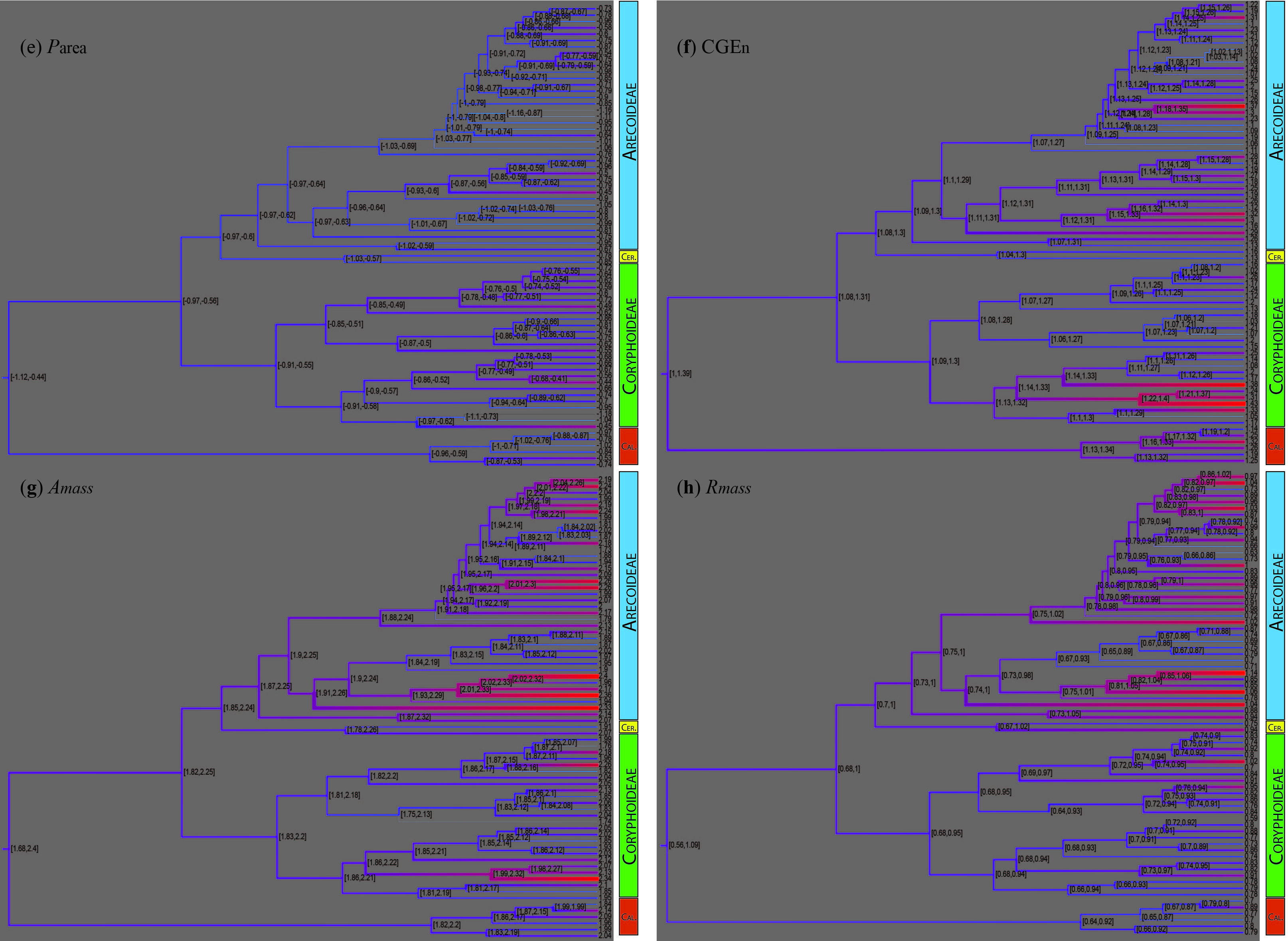
**

**
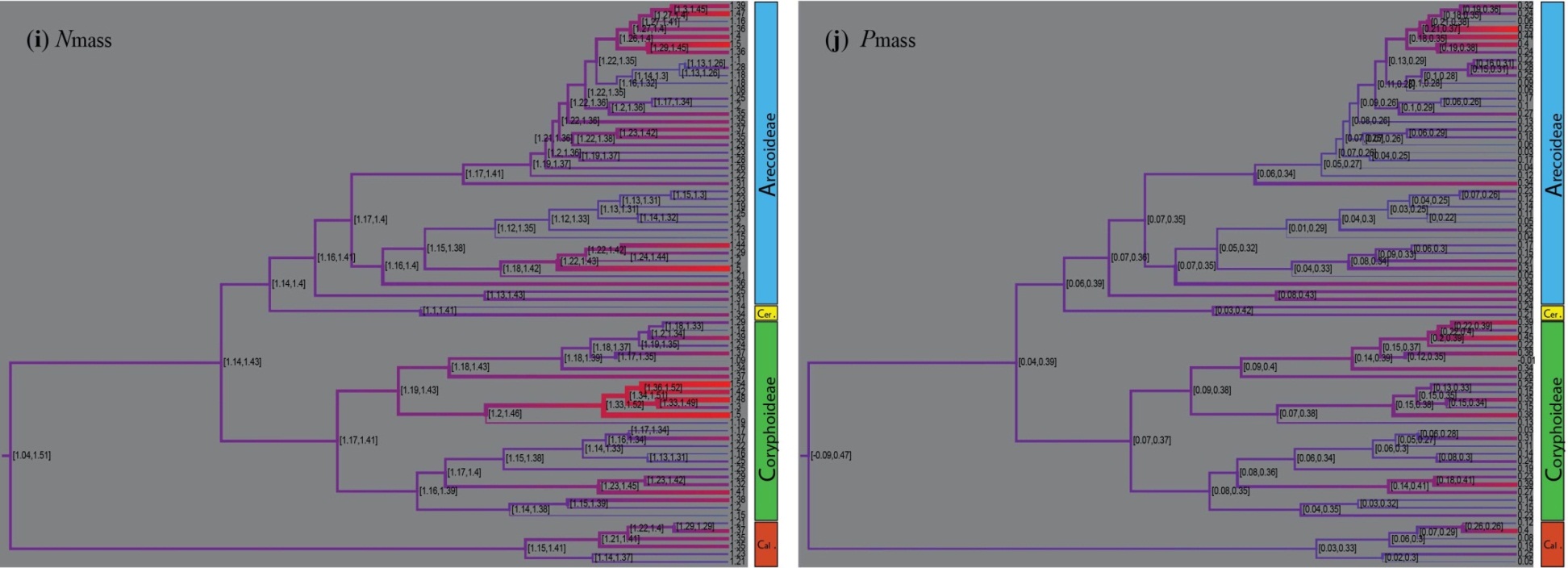
**

**S4 Fig. Maximum clade credibility (MCC) tree obtained with BEAST analysis of DNA and multivariate trait partitions.** Evolution of LES data is illustrated on this tree. Nodal values show ancestral 95% HPD interval for traits. Tips show trait values for extant species. Thickness of branches indicates a decrease, no change, or increase in trait value. Colours are added to aid in easy recognition of decrease, no change, or increase in trait values; low values = blue; high values = red.
