# Supplementary material for: Convergent Evolution towards High Net Carbon Gain Efficiency Contributes to the Shade Tolerance of Palms (Arecaceae)
Source: PLoS One. 2015 Oct 13;10(10):e0140384. doi: 10.1371/journal.pone.0140384 (PMC4604201; doi:10.1371/journal.pone.0140384)
Supplement: S1 Method — (DOCX) [file pone.0140384.s005.docx]

**S1 Method.**

**DNA sequences alignment and phylogenetic reconstructions of the palm species grown in the common garden.**

We used a combination of chloroplast and nuclear DNA markers to construct a phylogenetic tree following Baker et al. (2009, 2011) [1, 2]. In the study of Baker et al. (2009) [1], the four chloroplast gene regions (rbcL, rps16, matK and trnL-trnF) were shown to have particularly high levels of informative characters. In addition, Baker et al. (2011) [2] found that the two low-copy nuclear gene regions (prk and rpb2), were both highly informative when analyzing the phylogenetic relationships of Arecoideae, which is the largest and most diverse of the ﬁve subfamilies of palms.

DNA sequences were downloaded from published datasets of Baker et al. (2009, 2011) [1, 2] and GeneBank (<http://www.ncbi.nlm.nih.gov/genbank>), and their GenBank accession numbers provided in S1 Table. Mega 5 software [3] was used to align the sequences. Alignments of rbcL, rps16, matK and trnL-trnF were built upon Baker et al. (2009) [1] and alignments of prk and rpb2 were built upon Baker et al. (2011) [2], into which new sequences were incorporated manually. The six data partitions were aligned separately. Six Commelinid monocots (*Dasypogon*, *Fargesia*, *Hanguana*, *Kingia*, *Tradescantia* and *Zea*), were selected as out-groups following Baker et al. (2009) [1]. Additional sequences for these genera were obtained from Baker et al. (2009) [1] and GenBank.

Phylogenetic analyses were conducted using Bayesian Inference in MrBayes 3.22 [4] using model partitioning (all markers GTR+I+G) following model selection in jModeltest 2.1.4 [5].

MrBayes uses Markov chain Monte Carlo (MCMC) to approximate the posterior probabilities of trees [4, 6]. Four independent MCMC chains were run simultaneously and sampled every 100 generations for a total of 1x10^6^ generations. Upon completion, the standard deviation of split frequencies was below 0.001 indicating stationarity of the chains. We selected a burnin fraction of 25%, discarding the first 2500 saved trees, and a majority-rule consensus tree was produced. Seven species with ambiguous phylogenetic placement were removed from the dataset and the analysis was repeated with settings as before. All trees were read and modified using FigTree v1.40 [7]. The obtained phylogenetic tree contained 73 palm species (S1 Fig) with high bootstrap support and in accordance with the well-resolved palm phylogeny at generic levels [1].

**Supporting References**

1. Baker WJ, Savolainen V, Asmussen-Lange CB, Chase MW, Dransfield J, Forest F, et al. Complete generic-level phylogenetic analyses of palms (Arecaceae) with comparisons of supertree and supermatrix approaches. Syst Biol. 2009; 58: 240-256.

2. Baker WJ, Norup MV, Clarkson JJ, Couvreur TLP, Dowe JL, Lewis CE, et al. Phylogenetic relationships among arecoid palms (Arecaceae: Arecoideae). Ann Bot. 2011; 108: 1417-1432.

3. Tamura K, Peterson D, Peterson N, Stecher G, Nei M, Kumar S. Mega5: Molecular evolutionary genetics analysis using maximum likelihood, evolutionary distance, and maximum parsimony methods. Mol Biol Evol. 2011; 28: 2731-2739.

4. Ronquist F, Teslenko M, van der Mark P, Ayres DL, Darling A, Höhna S, et al. MrBayes 3.2: efficient bayesian phylogenetic inference and model choice across a large model space. Syst Biol. 2012; 61: 539-542.

5. Darriba D, Taboada GL, Doallo R, Posada D. jModelTest 2: more models, new heuristics and parallel computing. Nat Methods. 2012; 9: 772-772.

6. Huelsenbeck JP, Ronquist F. MrBayes: Bayesian inference of phylogenetic trees. Bioinformatics. 2001; 17: 754-755.

7. Rambaut A. FigTree: Tree figure drawing tool, v1.4.0. 2012. Available: <http://tree.bio.ed.ac.uk/software/figtree/>. Accessed 1 August 2013.
