# Supplementary material for: Convergent Evolution towards High Net Carbon Gain Efficiency Contributes to the Shade Tolerance of Palms (Arecaceae)
Source: PLoS One. 2015 Oct 13;10(10):e0140384. doi: 10.1371/journal.pone.0140384 (PMC4604201; doi:10.1371/journal.pone.0140384)
Supplement: S2 Method — (DOCX) [file pone.0140384.s006.docx]

**S2 Method.**

**Coevolutionary patterns of morphological traits and ancestral state reconstruction with simultaneous phylogenetic reconstruction.**

We used elements of the BEAST package (v1.8.2.-v2.0) [1] to simultaneously estimate phylogenetic relationships, reconstruct ancestral trait states and co-estimate continuous trait progression throughout our phylogeny. An xml-file with two data partitions was created with BEAUti (part of the BEAST package), employing a DNA data partition (9349 characters) and a multivariate trait partition (10 traits). Employed area- and mass based trait data was log_10_-transformed before analyses. We assigned a General Time Reversible (GTR) model to the DNA partition and a Homogenous Brownian Model to the multivariate trait data partition. We selected a Yule model of speciation for the tree model using a lognormal relaxed clock for the analysis. Initial analyses were run for 1x10^6^ generations to fine-tune operator settings, after which two independent analyses were run, each of 20x10^6^ generations. Results were checked using Tracer v1.6.0 [2]. We employed TreeAnnotator (part of the BEAST package) to produce an optimized maximum clade credibility (MCC) tree using mean target height and a burnin of 50%. FigTree v1.40 [3] was used to modify, edit and inspect the obtained MCC.

**Supporting References**

1. Bouckaert R, Heled J, Kühnert D, Vaughan T, Wu C-H, Xie D, et al. Beast 2: a software platform for Bayesian evolutionary analysis. PLoS Comp Biol. 2014; 10: e1003537.

2. Rambaut A, Suchard MA, Xie D, Drummond AJ. Tracer: MCMC trace analysis tool, v1.6.0. 2013. Available: <http://tree.bio.ed.ac.uk/software/tracer/>. Accessed 3 April 2015.

3. Rambaut A. FigTree: Tree figure drawing tool, v1.4.0. 2012. Available: <http://tree.bio.ed.ac.uk/software/figtree/>. Accessed 1 August 2013.
