# Supplementary material for: Convergent Evolution towards High Net Carbon Gain Efficiency Contributes to the Shade Tolerance of Palms (Arecaceae)
Source: PLoS One. 2015 Oct 13;10(10):e0140384. doi: 10.1371/journal.pone.0140384 (PMC4604201; doi:10.1371/journal.pone.0140384)
Supplement: S1 Table — (DOCX) [file pone.0140384.s007.docx]

**S1 Table. Description of the study palm species grown in common garden with their GenBank accession numbers for DNA sequences.** Species that occur naturally in high light or thrive in it in cultivation or some wild circumstances were identified by William J. Baker and were marked in bold font.

| Species | Tribe | Subfamily | Growth  form | Height  (m) | rbcL | rps16 | matK | trnL-trnF | rpb2 | prk |
| --- | --- | --- | --- | --- | --- | --- | --- | --- | --- | --- |
| 73 palm species included in phylogenetic generalized least squares analysis | | | | | | | | | | |
| ***Actinorhytis calapparia*** | Areceae | Arecoideae | Tree | 8.2 | AJ829847 | AM116814 | AM114659 | AM113658 | AJ830024 | AF453330 |
| *Aiphanes horrida* | Cocoseae | Arecoideae | Tree | 2.1 | AJ404831 | AJ404953 | AM114641 | HQ265761 | EF491155 | AY601211 |
| ***Allagoptera arenaria*** | Cocoseae | Arecoideae | Shrub | 1.3 | AJ404828 | AJ240902 | AM114635 | AJ241311 | AJ830152 | AF453331 |
| *Archontophoenix purpurea* | Areceae | Arecoideae | Tree | 3.7 | AJ404806 | AJ404937 | AM114660 | AJ404904 | AJ830028 | AJ831227 |
| *Areca triandra* | Areceae | Arecoideae | Tree | 5.8 | AJ404819 | AJ404945 | AM114664 | AJ404912 | FJ200370 | AY348912 |
| *Arenga hookeriana* | Caryoteae | Coryphoideae | Shrub | 1.9 | AJ404788 | AJ240882 | AM114592 | AJ241291 | AM903114 | AM900724 |
| *Astrocaryum murumuru* | Cocoseae | Arecoideae | Shrub | 3.8 | JX903251 | EU004910 | EU004872 | HQ265774 | HQ265635 | HQ265588 |
| ***Attalea speciosa*** | Cocoseae | Arecoideae | Tree | 4.2 | AJ404829 | AJ240903 | AM114636 | AJ241312 | AJ830207 | AY601246 |
| ***Bactris gasipaes*** | Cocoseae | Arecoideae | Tree | 9.5 | AM110214 | AM116806 | AM114642 | HQ265789 | HQ265650 | HQ265602 |
| *Beccariophoenix*  *madagascariensis* | Cocoseae | Arecoideae | Tree | 3.9 | AJ404826 | AJ404951 | AM114632 | AJ404918 | AJ830155 | AF453335 |
| *Bentinckia nicobarica* | Areceae | Arecoideae | Tree | 2.5 | AM110239 | AM116844 | AM114705 | AM113687 | AJ830032 | AJ831234 |
| ***Bismarckia nobilis*** | Borasseae | Coryphoideae | Tree | 5.3 | JX088664.1:  57164-58627 | JX088664.1:  5073-6189 | AM114597 | AM113634 | AM903123 | AM900729 |
| *Borassodendron*  *machadonis* | Borasseae | Coryphoideae | Tree | 5.5 | AJ404768 | AJ404927 | AM114603 | AJ404894 | AM903131 | AM900737 |
| ***Borassus flabellifer*** | Borasseae | Coryphoideae | Tree | 6.7 | AM110202 | AM116793 | AM114604 | AM113637 | AM903138 | FJ200377 |
| *Brassiophoenix schumannii* | Areceae | Arecoideae | Tree | 1.8 | AJ404815 | AJ240897 | AM114699 | AJ241306 | AJ830195 | AJ831235 |
| ***Butia capitata*** | Cocoseae | Arecoideae | Tree | 1.5 | JX903252 | EU004908 | EU004870 | EU004864 | EF491157 | AY601251 |
| *Calamus caesius* | Calameae | Calamoideae | Liana | 2.7 | AY044619 | AJ240870 | AM114551 | AJ241279 | AM903105 | AM900751 |
| *Calyptrocalyx forbesii* | Areceae | Arecoideae | Shrub | 1.9 | AM110232 | AM116834 | AM114687 | AM113677 | AJ830042 | AJ831246 |
| ***Carpentaria acuminata*** | Areceae | Arecoideae | Tree | 8.4 | AJ829858 | AM116840 | AM114697 | AM113683 | AJ830196 | AJ831259 |
| *Carpoxylon macrospermum* | Areceae | Arecoideae | Tree | 3.5 | AJ829859 | AM116824 | AM114673 | AM113667 | AJ830055 | AF453337 |
| *Caryota mitis* | Caryoteae | Coryphoideae | Shrub | 3.0 | AJ404790 | AJ240883 | AM114590 | AJ241292 | AJ830156 | AF453338 |
| *Chamaedorea seifrizii* | Chamaedoreeae | Arecoideae | Shrub | 1.8 | JX088667.1: 55806-57260 | JX088667.1: 4681-5779 | DQ178689 | JX088667.1: 47738-48699 | EF491124 | EF491084 |
| ***Chamaerops humilis*** | Trachycarpeae | Coryphoideae | Tree | 2.4 | AJ404754 | AM116777 | AM114568 | AJ241260 | AY543097 | AF453339 |
| *Chambeyronia macrocarpa* | Areceae | Arecoideae | Tree | 2.5 | AM110222 | AM116816 | AM114662 | AM113660 | AJ830056 | AJ831260 |
| *Chuniophoenix nana* | Chuniophoeniceae | Coryphoideae | Shrub | 1.6 | AJ404764 | AJ240860 | AM114587 | AJ241269 | AM903111 | AM900721 |
| ***Coccothrinax crinita*** | Cryosophileae | Coryphoideae | Tree | 1.5 | AJ404751 | AJ240848 | AM114558 | AJ241257 | EU215506 | EU215475 |
| ***Cocos nucifera*** | Cocoseae | Arecoideae | Tree | 13.1 | KF285453.1: 54542-55996 | KF285453.1: 4700-5819 | AM114637 | KF285453.1: 45933-46951 | EF491150 | AY601232 |
| ***Copernicia prunifera*** | Trachycarpeae | Coryphoideae | Tree | 1.4 | AM110199 | AM116785 | AM114582 | AM113630 | EU215513 | EU215482 |
| ***Corypha umbraculifera*** | Corypheae | Coryphoideae | Tree | 5.4 | AJ404761 | AJ240858 | AM114595 | AJ241267 | HQ720514 | AM900727 |
| *Cryosophila warscewiczii* | Cryosophileae | Coryphoideae | Tree | 2.6 | AJ404747 | AJ240846 | AM114563 | AJ241255 | EU215492 | EU215462 |
| *Cyrtostachys renda* | Areceae | Arecoideae | Shrub | 1.5 | AJ404810 | AJ404940 | AM114707 | AJ404907 | AJ830062 | AF453341 |
| *Daemonorops jenkinsiana* | Calameae | Calamoideae | Liana | 2.9 | AJ829866 | AJ242165 | - | - | - | - |
| *Desmoncus orthacanthos* | Cocoseae | Arecoideae | Liana | 2.3 | AM110215 | HQ265705 | AM114643 | HQ265800 | EF491156 | HQ265611 |
| *Dictyosperma album* | Areceae | Arecoideae | Tree | 5.1 | AM110241 | AM116846 | AM114708 | AM113689 | AJ830064 | AF453343 |
| *Drymophloeus hentyi* | Areceae | Arecoideae | Tree | 2.4 | AY012494 | - | FR832755 | - | JF833397 | JF833375 |
| ***Dypsis lutescens*** | Areceae | Arecoideae | Shrub | 3.5 | AJ404800 | AJ404934 | AM114681 | AJ404901 | AJ830078 | AF453346 |
| *Elaeis guineensis* | Cocoseae | Arecoideae | Tree | 4.3 | JF274081.1:  56511-57965 | JF274081.1: 4840-5956 | AM114644 | JF274081.1: 47500-48557 | AJ830163 | AY601219 |
| *Euterpe oleracea* | Euterpeae | Arecoideae | Shrub | 3.9 | AJ404802 | AJ240889 | AM114647 | AJ241298 | - | AF453347 |
| *Hydriastele microspadix* | Areceae | Arecoideae | Shrub | 3.2 | AJ404817 | AJ404943 | AM114712 | AJ404910 | AY543136 | AY348932 |
| *Hyophorbe lagenicaulis* | Chamaedoreeae | Arecoideae | Tree | 1.9 | AJ404785 | AJ240879 | AM114620 | AJ241288 | AJ830168 | AF453351 |
| ***Hyphaene thebaica*** | Borasseae | Coryphoideae | Tree | 4.2 | AJ404770 | AJ240865 | AM114599 | AJ241274 | AM903127 | AM900733 |
| *Iguanura wallichiana* | Areceae | Arecoideae | Shrub | 0.5 | AJ404820 | AJ404946 | AM114714 | AJ404913 | AY543099 | AF453352 |
| *Kentiopsis oliviformis* | Areceae | Arecoideae | Tree | 2.5 | AJ404809 | AJ240892 | AM114663 | AJ241788 | AY543100 | AF453353 |
| *Kerriodoxa elegans* | Chuniophoeniceae | Coryphoideae | Shrub | 2.1 | AJ404765 | AJ240861 | AM114588 | AJ241270 | HQ720523 | AJ831355 |
| *Latania verschaffeltii* | Borasseae | Coryphoideae | Tree | 3.0 | AF829878 | AM116792 | AM114601 | AM113636 | AM903144 | AM900750 |
| *Lytocaryum weddellianum* | Cocoseae | Arecoideae | Tree | 1.5 | AY044633 | - | FR832789 | - | JQ821999 | JQ821975 |
| ***Mauritia flexuosa*** | Lepidocaryeae | Calamoideae | Tree | 3.8 | AJ404777 | AJ240872 | AM114545 | AJ241281 | - | - |
| ***Nannorrhops ritchiana*** | Chuniophoeniceae | Coryphoideae | Shrub | 1.5 | AJ404763 | AJ240859 | AM114589 | AJ241268 | AM903112 | AM900722 |
| *Normanbya normanbyi* | Areceae | Arecoideae | Tree | 2.2 | AJ829890 | - | - | - | AJ830198 | AF453363 |
| ***Phoenix dactylifera*** | Phoeniceae | Coryphoideae | Tree | 6.6 | GU811709.2:  56909-58363 | GU811709.2: 4870-5989 | GU811709.2: 1739-3283 | GU811709.2: 47962-48967 | - | - |
| *Phytelephas aequatorialis* | Phytelepheae | Ceroxyloideae | Tree | 3.5 | AJ404835 | AJ240908 | AM114613 | AJ241317 | AJ830178 | AJ831361 |
| *Pinanga coronata* | Areceae | Arecoideae | Shrub | 3.5 | AJ829898 | - | - | - | AY543156 | AY348944 |
| *Plectocomia elongata* | Calameae | Calamoideae | Liana | 2.6 | AY298848 | AJ242167 | AM114550 | AM113617 | - | - |
| *Ptychosperma macarthurii* | Areceae | Arecoideae | Shrub | 2.5 | AM110235 | AM116838 | AM114693 | AM113681 | AJ830201 | AJ831325 |
| ***Raphia farinifera*** | Lepidocaryeae | Calamoideae | Tree | 7.6 | AJ829907 | AJ242184 | AM114544 | AM113612 | - | - |
| *Ravenea rivularis* | Ceroxyleae | Ceroxyloideae | Tree | 2.7 | AJ404783 | AJ240877 | AM114610 | AJ241286 | EF128416 | EF128379 |
| *Rhapidophyllum hystrix* | Trachycarpeae | Coryphoideae | Shrub | 1.2 | AJ404753 | AM116778 | AM114571 | AJ241259 | HQ720571 | EU215458 |
| *Rhapis excelsa* | Trachycarpeae | Coryphoideae | Shrub | 1.7 | AJ404756 | AJ240853 | AM114573 | AJ241262 | HQ720572 | - |
| *Roystonea oleracea* | Roystoneae | Arecoideae | Tree | 8.3 | AJ404805 | AJ404936 | AM114630 | AJ404903 | AJ830184 | AJ831372 |
| ***Sabal minor*** | Sabaleae | Coryphoideae | Shrub | 1.0 | AM110191 | AM116770 | AM114554 | AM113618 | - | EF667948 |
| *Salacca zalacca* | Calameae | Calamoideae | Shrub | 4.4 | AY012472 | AJ242176 | AM114547 | AM113614 | - | - |
| *Satakentia liukiuensis* | Areceae | Arecoideae | Tree | 2.9 | AM110227 | AM116825 | AM114674 | AM113668 | AJ830146 | AF453376 |
| *Schippia concolor* | Cryosophileae | Coryphoideae | Tree | 2.2 | AJ404749 | AJ404924 | AM114555 | AJ404891 | EU215486 | EU215457 |
| *Serenoa repens* | Trachycarpeae | Coryphoideae | Shrub | 1.5 | AJ404760 | AM116788 | AM114585 | AJ241266 | HQ720585 | EU215464 |
| *Syagrus romanzoffiana* | Cocoseae | Arecoideae | Tree | 7.2 | AJ404827 | AJ240901 | AM114638 | AJ241310 | AY779378 | AY601248 |
| *Thrinax radiata* | Cryosophileae | Coryphoideae | Tree | 1.5 | AJ404750 | AM116774 | AM114561 | AJ241256 | EU215495 | EU215460 |
| ***Trachycarpus martianus*** | Trachycarpeae | Coryphoideae | Tree | 1.7 | JX903256 | AJ404925 | AM114570 | AJ404892 | - | - |
| *Trithrinax acanthocoma* | Cryosophileae | Coryphoideae | Tree | 1.3 | AY298852 | AJ240844 | AM114556 | AJ241253 | - | - |
| *Veitchia filifera* | Areceae | Arecoideae | Tree | 5.1 | AY012496 | AJ404942 | AM114696 | AJ404909 | AJ830205 | AJ831342 |
| *Verschaffeltia splendida* | Areceae | Arecoideae | Tree | 3.5 | AJ829916 | - | - | - | AJ830150 | AF453381 |
| *Wallichia disticha* | Caryoteae | Coryphoideae | Tree | 4.3 | AJ404792 | AJ240884 | AM114594 | AJ241293 | GU929697 | - |
| ***Washingtonia robusta*** | Trachycarpeae | Coryphoideae | Tree | 5.5 | AM110201 | AM116789 | AM114586 | AM113633 | HQ720593 | - |
| ***Wodyetia bifurcata*** | Areceae | Arecoideae | Tree | 4.8 | AM110236 | AM116841 | AM114698 | AM113684 | AJ830206 | AJ831343 |
| 7 palm species excluded from the phylogenetic generalized least squares analysis | | | | | | | | | | |
| ***Acoelorrhaphe wrightii*** | Trachycarpeae | Coryphoideae | Tree | 1.8 | AM110197 | AM116782 | AM114579 | AM113627 | HQ720485 | EU215477 |
| *Johannesteijsmannia altifrons* | Trachycarpeae | Coryphoideae | Shrub | 1.5 | AJ404758 | AJ240855 | AM114576 | AJ241264 | HQ720517 | - |
| *Licuala grandis* | Trachycarpeae | Coryphoideae | Tree | 1.8 | AY012462 | AJ240856 | AM114575 | AJ241265 | HQ720529 | - |
| *Livistona chinensis* | Trachycarpeae | Coryphoideae | Tree | 4.1 | AJ404757 | AJ240854 | AM114574 | AJ241263 | HQ720541 | - |
| *Oenocarpus bacaba* | Euterpeae | Arecoideae | Tree | 2.6 | JQ626213 | AM116782 | JQ626537 | - | - | - |
| *Caryota urens* | Caryoteae | Coryphoideae | Tree | 6.5 | JF344863 | - | JF344998 | AB817480 | FJ200375 | - |
| *Chuniophoenix hainanensis* | Chuniophoeniceae | Coryphoideae | Tree | 2.5 | AM903192 | - | - | AM903232 | AM903110 | - |

DNA sequences were downloaded from Baker et al. (2009, 2011) [1, 2] and GeneBank ([http://www.ncbi.nlm.nih.gov/genbank)](http://www.ncbi.nlm.nih.gov/genbank)%20and).

**References**

1. Baker WJ, Savolainen V, Asmussen-Lange CB, Chase MW, Dransfield J, Forest F, et al. Complete generic-level phylogenetic analyses of palms (Arecaceae) with comparisons of supertree and supermatrix approaches. Syst Biol. 2009; 58: 240-256.

2. Baker WJ, Norup MV, Clarkson JJ, Couvreur TLP, Dowe JL, Lewis CE, et al. Phylogenetic relationships among arecoid palms (Arecaceae: Arecoideae). Ann Bot. 2011; 108: 1417-1432.
