# Supplementary material for: Convergent Evolution towards High Net Carbon Gain Efficiency Contributes to the Shade Tolerance of Palms (Arecaceae)
Source: PLoS One. 2015 Oct 13;10(10):e0140384. doi: 10.1371/journal.pone.0140384 (PMC4604201; doi:10.1371/journal.pone.0140384)
Supplement: S2 Table — (DOCX) [file pone.0140384.s008.docx]

**S2** **Table. Compiled data on leaf traits of field palm species.**

| Data source  references | Species | Tribe | Subfamily | Growth  form | LMA | *A*_area_ | *R*_area_ | *N*_area_ | *P*_area_ | CGE_n_ | *A*_mass_ | *R*_mass_ | *N*_mass_ | *P*_mass_ | Note |
| --- | --- | --- | --- | --- | --- | --- | --- | --- | --- | --- | --- | --- | --- | --- | --- |
| [1] | *Chamaedorea elegans* | Chamaedoreeae | Arecoideae | Shrub | 44.3 | 1.15 | 0.08 | 1.15 |  | 14.29 | 25.96 | 1.81 | 26.0 |  | *1 |
| [2] | *Astrocaryum mexicanum* | Cocoseae | Arecoideae | Tree | 74.8 |  |  | 1.25 | 0.119 |  |  |  | 16.69 | 1.59 |  |
| [2] | *Bactris trichophylla* | Cocoseae | Arecoideae | Shrub | 54.8 |  |  | 0.84 | 0.059 |  |  |  | 15.38 | 1.08 |  |
| [2] | *Chamaedorea alternans* | Chamaedoreeae | Arecoideae | Tree | 41.9 |  |  | 0.74 | 0.059 |  |  |  | 17.65 | 1.41 |  |
| [2] | *Chamaedorea pinnatifrons* | Chamaedoreeae | Arecoideae | Tree | 40.9 |  |  | 0.90 | 0.085 |  |  |  | 22.03 | 2.07 |  |
| [3, 4] | *Asterogyne martiana* | Geonomateae | Arecoideae | Shrub | 66.1 | 4.14 | 0.14 | 1.21 |  | 30.22 | 62.72 | 2.08 | 18.28 |  | *2 |
| [3, 4] | *Bactris spp* | Cocoseae | Arecoideae | Shrub | 86.2 | 4.86 | 0.22 | 1.51 |  | 21.79 | 56.38 | 2.59 | 17.46 |  |  |
| [3, 4] | *Cryosophila warscewiczii* | Cryosophileae | Coryphoideae | Tree | 69.9 | 4.09 | 0.28 | 2.49 | 0.098 | 14.77 | 58.49 | 3.96 | 35.74 | 1.40 |  |
| [3, 4] | *Euterpe precatoria* | Euterpeae | Arecoideae | Tree | 58.1 | 1.68 | 0.17 | 1.00 | 0.044 | 9.66 | 28.90 | 2.99 | 17.25 | 0.75 |  |
| [3, 4] | *Geonoma congesta* | Geonomateae | Arecoideae | Shrub | 74.8 | 5.16 | 0.43 | 1.35 | 0.053 | 11.99 | 69.68 | 5.77 | 18.02 | 0.74 |  |
| [3, 4] | *Geonoma cuneata* | Geonomateae | Arecoideae | Shrub | 62.8 | 2.67 | 0.43 | 1.16 | 0.072 | 6.27 | 42.25 | 6.67 | 18.41 | 1.14 |  |
| [3, 4] | *Geonoma spp* | Geonomateae | Arecoideae | Shrub | 50.1 | 2.82 | 0.13 | 1.06 | 0.049 | 21.90 | 69.37 | 2.47 | 21.47 | 0.98 |  |
| [3, 4] | *Iriartea deltoidea* | Iriarteeae | Arecoideae | Tree | 113.6 | 11.2 | 0.76 | 2.23 | 0.138 | 14.76 | 98.56 | 6.68 | 19.55 | 1.21 |  |
| [3, 4] | *Prestoea decurrens* | Euterpeae | Arecoideae | Tree | 82.0 | 5.06 | 0.29 | 1.70 | 0.074 | 17.27 | 61.73 | 3.57 | 20.66 | 0.90 |  |
| [3, 4] | *Socratea exorrhiza* | Iriarteeae | Arecoideae | Tree | 63.7 | 12.7 | 0.56 | 1.29 | 0.057 | 22.77 | 199.9 | 8.78 | 20.17 | 0.90 |  |
| [3, 4] | *unknown palm species* |  |  | Shrub | 69.5 | 3.26 | 0.15 | 1.26 |  | 22.10 | 38.47 | 2.03 | 18.96 |  |  |
| [3, 4] | *Welfia regia* | Geonomateae | Arecoideae | Tree | 102.3 | 5.13 | 0.34 | 1.81 | 0.088 | 15.02 | 56.43 | 3.28 | 15.88 | 0.77 |  |
| [5] | *Asterogyne martiana* | Geonomateae | Arecoideae | Shrub | 42.2 | 3.26 | 0.18 | 0.71 |  | 18.11 | 78.1 | 4.27 | 16.9 |  | *3 |
| [5] | *Geonoma cuneata* | Geonomateae | Arecoideae | Shrub | 47.1 | 3.79 | 0.10 | 0.85 |  | 37.90 | 81.7 | 2.12 | 18.0 |  |  |
| [5] | *Geonoma congesta* | Geonomateae | Arecoideae | Shrub | 38.2 | 3.08 | 0.14 | 0.78 |  | 22.00 | 81.7 | 3.66 | 20.3 |  |  |
| [6] | *Calamus caryotoides* | Calameae | Calamoideae | Liana | 167.0 |  |  | 2.82 |  |  |  |  | 16.9 |  |  |
| [6] | *Calamus australis* | Calameae | Calamoideae | Liana | 58.8 |  |  | 1.16 |  |  |  |  | 19.8 |  |  |
| [6] | *Geonoma congesta* | Geonomateae | Arecoideae | Shrub | 70.9 |  |  | 0.90 |  |  |  |  | 12.7 |  |  |
| [6] | *Welfia regia* | Geonomateae | Arecoideae | Tree | 62.1 |  |  | 0.82 |  |  |  |  | 13.2 |  |  |
| [7] | *Socratea exorrhiza* | Iriarteeae | Arecoideae | Tree | 150.5 |  |  | 2.68 | 0.185 |  |  |  | 17.80 | 1.23 | *4 |
| [7] | *Iriartea deltoidea* | Iriarteeae | Arecoideae | Tree | 108.0 |  |  | 1.70 | 0.136 |  |  |  | 15.73 | 1.26 |  |
| [8] | *Livistona humilis* | Trachycarpeae | Coryphoideae | Tree | 235.6 |  |  | 2.66 |  |  |  |  | 11.29 |  | *5 |
| [8] | *Livistona humilis* | Trachycarpeae | Coryphoideae | Tree | 200.8 |  |  | 3.51 |  |  |  |  | 17.50 |  |  |
| [9] | *Licuala ramsayi* | Trachycarpeae | Coryphoideae | Tree | 48 | 2.1 | 0.09 | 0.76 | 0.04 | 24.46 | 53.4 | 1.79 | 16.8 | 0.8 |  |
| [9] | *Linospadix minor* | Areceae | Arecoideae | Shrub | 44 | 2.7 | 0.17 | 0.78 | 0.04 | 16.21 | 61.8 | 3.78 | 17.7 | 1.0 |  |

LMA (g m^-2^), leaf mass per area; *A*_area_ (umol s^-1^ m^-2^) and *A*_mass_ (nmol s^-1^ g^-1^), area- and mass-based maximum photosynthetic rate; *R*_area_ (umol s^-1^ m^-2^) and *R*_mass_ (nmol s^-1^ g^-1^), area- and mass-based dark respiration; *N*_area_ (g m^-2^) and *N*_mass_ (mg g^-1^), area- and mass-based nitrogen concentration; *P*_area_ (g m^-2^) and *P*_mass_ (mg g^-1^), area- and mass-based phosphorus concentration; CGE_n_ (unitless), net carbon gain efficiency, determined as *A*_area_ divided by *R*_area._

Notes:*1: *R*_area_ and CGE_n_ was calculated according to *R*_area_ = 0.07*A*_area_; *2: original data were processed according to the maximum *A*_area_; *3: species grown in full-shade environment were used; *4: mean values of different height classes; *5: same species in different sites and we got the data from Wright et al. (2004) [10].

**Data source references**

1. Anten NPR, Ackerly DD. Canopy-level photosynthetic compensation after defoliation in a tropical understorey palm. Funct Ecol. 2001; 15: 252-262.

2. Bongers F, Popma J. Leaf characteristics of the tropical rain forest flora of Los Tuxtlas, Mexico. Bot Gaz. 1990; 151: 354-365.

3. Cavaleri MA, Oberbauer SF, Ryan MG. Foliar and ecosystem respiration in an old-growth tropical rain forest. Plant, Cell Environ. 2008; 31: 473-483.

4. Cavaleri MA, Oberbauer SF, Clark DB, Clark DA, Ryan MG. Height is more important than light in determining leaf morphology in a tropical forest. Ecology. 2010; 91: 1730-1739.

5. Chazdon RL. Light variation and carbon gain in rain forest understorey palms. J Ecol. 1986; 74: 995-1012.

6. Grubb PJ, Jackson RV, Barberis IM, Bee JN, Coomes DA, Dominy NJ, et al. Monocot leaves are eaten less than dicot leaves in tropical lowland rain forests: correlations with toughness and leaf presentation. Ann Bot. 2008; 101: 1379-1389.

7. Rich PM, Holbrook NM, Luttinger N. Leaf development and crown geometry of two Iriarteoid palms. Am J Bot. 1995; 82: 328-336.

8. Schulze ED, Williams RJ, Farquhar GD, Schulze W, Langridge J, Miller JM, et al. Carbon and nitrogen isotope discrimination and nitrogen nutrition of trees along a rainfall gradient in northern Australia. Aust J Plant Physiol. 1998; 25: 413-425.

9. Weerasinghe LK, Creek D, Crous KY, Xiang S, Liddell MJ, Turnbull MH, et al. Canopy position affects the relationships between leaf respiration and associated traits in a tropical rainforest in Far North Queensland. Tree Physiol. 2014; 34: 564-584.

10. Wright IJ, Reich PB, Westoby M, Ackerly DD, Baruch Z, Bongers F, et al. The worldwide leaf economics spectrum. Nature. 2004; 428: 821-827.
