# Supplementary material for: Convergent Evolution towards High Net Carbon Gain Efficiency Contributes to the Shade Tolerance of Palms (Arecaceae)
Source: PLoS One. 2015 Oct 13;10(10):e0140384. doi: 10.1371/journal.pone.0140384 (PMC4604201; doi:10.1371/journal.pone.0140384)
Supplement: S3 Table — (DOCX) [file pone.0140384.s009.docx]

**S3 Table. Mean values and ranges for 10 leaf traits of 80 common garden palm species.**

| Leaf traits | Mean | Minimum | Maximum |
| --- | --- | --- | --- |
| LMA (g m^-2^) | 111.2 | 40.6 | 227.3 |
| *A*_area_ (umol s^-1^ m^-2^) | 11.6 | 4.03 | 22.2 |
| *R*_area_ (umol s^-1^ m^-2^) | 0.75 | 0.29 | 1.51 |
| *N*_area_ (g m^-2^) | 2.07 | 0.96 | 3.72 |
| *P*_area_ (g m^-2^) | 0.18 | 0.07 | 0.36 |
| CGE_n_ (unitless) | 15.8 | 10.5 | 26.9 |
| *A*_mass_ (nmol s^-1^ g^-1^) | 113.1 | 49.2 | 251.6 |
| *R*_mass_ (nmol s^-1^ g^-1^) | 7.11 | 3.87 | 13.8 |
| *N*_mass_ (mg g^-1^) | 19.6 | 12.1 | 34.5 |
| *P*_mass_ (mg g^-1^) | 1.66 | 0.75 | 3.53 |

See S2 Table for trait abbreviations.
