# Supplementary material for: Convergent Evolution towards High Net Carbon Gain Efficiency Contributes to the Shade Tolerance of Palms (Arecaceae)
Source: PLoS One. 2015 Oct 13;10(10):e0140384. doi: 10.1371/journal.pone.0140384 (PMC4604201; doi:10.1371/journal.pone.0140384)
Supplement: S4 Table — (DOCX) [file pone.0140384.s010.docx]

**S4 Table. Differences in slope and intercept of bivariate relationships of field palms with those of the global dataset and dicotyledonous broad-leaved trees in tropical rain forests (dicot TRF trees)**.

| Y - X | Dataset | Slope | Intercept |
| --- | --- | --- | --- |
| *A*_area_ - *R*_area_ | global dataset | ns | *** |
|  | dicot TRF trees | ns | Ns |
| *N*_area_ - LMA | global dataset | *** | - |
|  | dicot TRF trees | ns | Ns |
| *P*_area_ - LMA | global dataset | * | - |
|  | dicot TRF trees | ns | Ns |
| *R*_area_ - LMA | global dataset | *** | - |
|  | dicot TRF trees | - | - |
| *A*_area_ - *N*_area_ | global dataset | ns | *** |
|  | dicot TRF trees | ns | *** |
| *A*_area_ - *P*_area_ | global dataset | ** | - |
|  | dicot TRF trees | ns | * |
| *R*_area_ - *N*_area_ | global dataset | * | - |
|  | dicot TRF trees | ns | *** |
| *R*_area_ - *P*_area_ | global dataset | ** | - |
|  | dicot TRF trees | ns | Ns |

See S2 Table for trait abbreviations. ns: *P* > 0.05, * *P* < 0.05; ** *P* < 0.01; *** *P* < 0.001. All variables were log_10_-transformed and data were fitted by standardized major axis (SMA) regression using the SMATR package [1].

**References**

1. Warton DI, Wright IJ, Falster DS, Westoby M. Bivariate line-fitting methods for allometry. Biol Rev. 2006; 81: 259-291.
