# Supplementary material for: Convergent Evolution towards High Net Carbon Gain Efficiency Contributes to the Shade Tolerance of Palms (Arecaceae)
Source: PLoS One. 2015 Oct 13;10(10):e0140384. doi: 10.1371/journal.pone.0140384 (PMC4604201; doi:10.1371/journal.pone.0140384)
Supplement: S5 Table — (DOCX) [file pone.0140384.s011.docx]

**S5 Table. Correlation coefficients *r* and *P* values for each bivariate trait relationship fitted via phylogenetic generalized least squares (PGLS) framework.**

| y-x | *r* | *P* | y-x | *r* | *P* |
| --- | --- | --- | --- | --- | --- |
| *A*_area_-*R*_area_ | 0.83 | 0.000 |  |  |  |
| *N*_area_-LMA | 0.79 | 0.000 | *N*_mass_-LMA | -0.56 | 0.000 |
| *P*_area_-LMA | 0.77 | 0.000 | *P*_mass_-LMA | -0.31 | 0.003 |
| *A*_area_-LMA | 0.56 | 0.001 | *A*_mass_-LMA | -0.57 | 0.000 |
| *R*_area_-LMA | 0.75 | 0.000 | *R*_mass_-LMA | -0.52 | 0.000 |
| CGE_n_-LMA | -0.30 | 0.004 |  |  |  |
| *A*_area_-*N*_area_ | 0.69 | 0.000 | *A*_mass_-*N*_mass_ | 0.65 | 0.000 |
| *A*_area_-*P*_area_ | 0.58 | 0.000 | *A*_mass_-*P*_mass_ | 0.41 | 0.000 |
| *R*_area_-*N*_area_ | 0.80 | 0.000 | *R*_mass_-*N*_mass_ | 0.65 | 0.000 |
| *R*_area_-*P*_area_ | 0.73 | 0.000 | *R*_mass_-*P*_mass_ | 0.47 | 0.000 |

See S2 Table for trait abbreviations.
