# Supplementary material for: Convergent Evolution towards High Net Carbon Gain Efficiency Contributes to the Shade Tolerance of Palms (Arecaceae)
Source: PLoS One. 2015 Oct 13;10(10):e0140384. doi: 10.1371/journal.pone.0140384 (PMC4604201; doi:10.1371/journal.pone.0140384)
Supplement: S6 Table — (DOCX) [file pone.0140384.s012.docx]

**S6 Table. Results of the phylogenetic signal tests for area-based and mass-based LES traits among palm species.**

| Leaf traits | λ |
| --- | --- |
| **Area-based** | |
| LMA | 0.807 |
| *A*_area_ | 0.491 |
| *R*_area_ | 0.762 |
| *N*_area_ | 0.901 |
| *P*_area_ | 0.488 |
| **Mass-based** | |
| *A*_mass_ | 0.043 |
| *R*_mass_ | 0.212 |
| *N*_mass_ | 0.472 |
| *P*_mass_ | 0.000 |
|  |  |
| CGE_n_ (unitless) | 0.255 |

See S2 Table for trait abbreviations. Pagel’s λ [1] was calculated on non-transformed data.

**References**

1. Pagel M. Inferring the historical patterns of biological evolution. Nature. 1999; 401: 877-884.
